# Beyond Contagion: Reality Mining Reveals Complex Patterns of Social Influence

**DOI:** 10.1371/journal.pone.0135740

**Published:** 2015-08-27

**Authors:** Aamena Alshamsi, Fabio Pianesi, Bruno Lepri, Alex Pentland, Iyad Rahwan

**Affiliations:** 1 Department of Electrical Engineering and Computer Science, Masdar Institute of Science & Technology, Abu Dhabi, United Arab Emirates; 2 Foundation Bruno Kessler, Trento, Italy; 3 Media Laboratory, Massachusetts Institute of Technology, Cambridge, Massachusetts, United States of America; University of Waterloo, CANADA

## Abstract

Contagion, a concept from epidemiology, has long been used to characterize social influence on people’s behavior and affective (emotional) states. While it has revealed many useful insights, it is not clear whether the contagion metaphor is sufficient to fully characterize the complex dynamics of psychological states in a social context. Using wearable sensors that capture daily face-to-face interaction, combined with three daily experience sampling surveys, we collected the most comprehensive data set of personality and emotion dynamics of an entire community of work. From this high-resolution data about actual (rather than self-reported) face-to-face interaction, a complex picture emerges where contagion (that can be seen as adaptation of behavioral responses to the behavior of other people) cannot fully capture the dynamics of transitory states. We found that social influence has two opposing effects on states: *adaptation* effects that go beyond mere contagion, and *complementarity* effects whereby individuals’ behaviors tend to complement the behaviors of others. Surprisingly, these effects can exhibit completely different directions depending on the stable personality or emotional dispositions (stable traits) of target individuals. Our findings provide a foundation for richer models of social dynamics, and have implications on organizational engineering and workplace well-being.

## Introduction

Social influence is a fundamental force in society that drives the formation and propagation of opinions [1], attitudes [2], behaviors [3], social norms [4] and of psychological states [5]. Its power can be exploited to increase political participation [6], promote physical activity and personal well-being [7], and reduce energy consumption [8].

The metaphor of *contagion* provides a powerful framework for modeling social influence [9–11]. Psychological and behavioral phenomena can be seen to *spread*, like a disease, from one person to another as a result of face-to-face [12] or electronic communication [13, 14]. Recent work showed that contagion can characterize (at least partially) the spread of obesity [12], eating habits [15], cooperative behavior [16–18], generosity [19], smoking [20], happiness [21], smiling [22], depression [23, 24], and emotion more generally [25, 26]. It is also possible to estimate parameters of epidemic models, such as the Susceptible-Infected-Susceptible (SIS) model, directly from behavioral data [27, 28].

Recently, the notion of behavioral contagion in social networks has become a subject of heated debate, particularly surrounding the difficulty of differentiating between contagion and homophily from observational data [11, 29–31]. The present article is not a contribution to this debate, but rather raises a number of orthogonal issues that we believe are equally important to our understanding of social dynamics.

Firstly, existing literature has mostly captured the dynamics of long-lasting state, with a temporal resolution of months [10] or years [11], with no consideration to the fleeting states that we all go through daily. One possible reason for this could be that traditional sampling methodologies have so far made it difficult to capture those states, as well as the similarly fast-changing situational factors, at a high enough temporal resolution.

Another limitation of existing work stems from the fact that social and biological contagion are fundamentally different [32]. In particular, existing contagion models focus on social-situational influences [21, 27] and neglect the role of individual differences in psychological state dynamics [33, 34]. Individuals differ in their tendencies to experience given psychological states, since the *dynamics of states* are a result of the interplay between situational factors (e.g., social interaction) and individuals’ *stable traits* (also known as *dispositions*) [35]. Thus, one should expect that individual between-subject differences should play an equally important role in social dynamics as situational factors [36, 37].

For example, consider the Big-Five personality model, which measures openness, conscientiousness, extraversion, agreeableness, and neuroticism [38]. A person might be high on the extraversion *trait*, but his extraversion *state* fluctuates over time. Indeed, through experience sampling, Fleeson [39] showed that: (a) people pass through all levels of personality states in their daily lives; (b) the central tendency of personality states is stable and reflects the corresponding trait level; (c) within-person variation in personality states can be attributed to the interplay between situational aspects and the stable dispositions captured by traits.

Another source of limitation in the classical contagion model is that it typically deals with transition among discrete conditions—infected and not infected. Yet, sociological and psychological theory has long recognized that social influence often involves changes across continuous [40] or ordinal state structure [36], as people transition among levels of the same state (e.g. higher or lower extraversion).

We collected high-resolution data of daily face-to-face interaction among members of an organization, along with detailed experience sampling (3 times per day) of their Big-Five personality states, and their affective (emotional) states using measures of Positive and Negative Affect [41]. Results reveal a complex picture whereby: (a) contagion plays a marginal role; (b) more nuanced effects like *attraction*, *inertia*, *repulsion* and *push* that are reminiscent of the mimicry/adaptation vs. complementarity distinction [42, 43]; (c) these processes are moderated by individual dispositions (traits).

Our findings suggest that the intuitive preventive actions, of avoiding infection by staying away from people in undesirable states (e.g. depression) and seeking people in desirable states (e.g. high positive affect) may not always be effective. A more nuanced approach, which takes into account these subtle social influences, would be necessary. These results are highly relevant to organizational engineering [44] and efforts to make work environments, teamwork, and schools more effective [45–48].

## Methods

High-resolution sensors have made collecting and analyzing enormous amount of social interaction data possible [49–53], alleviating the exclusive reliance on unreliable, subjective self-reports based on people’s memory [54]. Moreover, sensors can log data at very fine time-scales without interfering with people’s routines or consuming their time, making it easier to investigate short-duration phenomena.

### Data Collection

**Fig 1:**
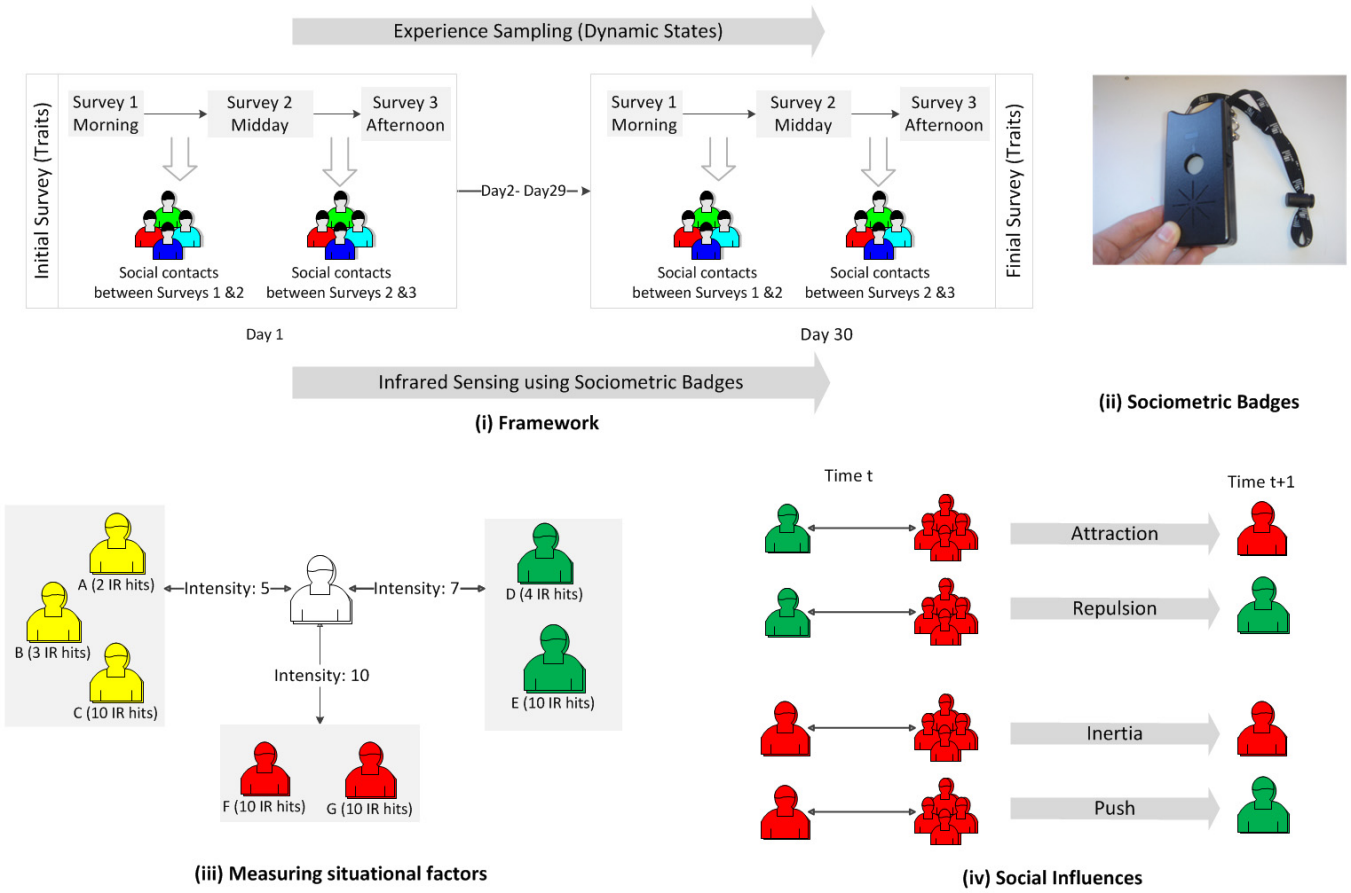
**(i) Framework of the Study:** First, participants filled a survey capturing personality and affect (stable) traits. Then they filled 3 daily surveys for 30 work days to measure personality and affect (dynamic) states. **(ii) Sociometric Badge:** Each participant’s social network between any two consecutive surveys was constructed from infrared sensor data from sociometric badges worn around the neck [49]. **(iii) Measuring Situational Factors:** An example of how the situational factors (intensity of contacts) is calculated. The ego’s infrared sensor (in the middle) detected 7 alters between two consecutive surveys. Two of the alters were in the high level (green) with 14 infrared hits, leading to intensity of contact 14/2 = 7. Similarly, the intensity of contact with three alters in the neutral level (yellow) is 15/3 = 5 and that for alters in the low level (red) is 20/2 = 10. **(iv) Social Influences:** Four possible social influences exemplified. Attraction: an ego in the high level interacts with others in the low level, then moves to the low level to adapt to his peers. Repulsion: a participant in the high level interacts with others in the low level, and consequently remains in the high level in complement to his peers. Inertia: a participant in the low level interacts with others in the same level, who prevent him from moving to a different level, maintaining his adaptation to their level. Push: a participant in the low level interacts with others in the same level, as a result pushing him away to a different, complementary level.

*Sociometric Badges*, designed and built by author Pentland, are capable of tracking various activities and behaviors of individuals [49]. These sensors –Fig 1(ii)– track face-to-face interactions by means of infrared (IR) sensors that recognize similar sensors facing them, implying that the two participants wearing them had a conversation or eye contact.

We used these badges to track face-to-face interactions of 52 individuals and conducted three daily experience sampling surveys [55] to collect information about their personality and emotional states –Fig 1(i). In addition, affect and personality (stable) traits were measured at the beginning of the study. This methodology was applied to five personality states and their corresponding traits (extraversion, agreeableness, conscientiousness, emotional stability and creativity) and two affect states and their corresponding traits –high positive affect (HPA) and low negative affect (LNA).

Specifically, we followed the well-established procedure proposed by Fleeson [39]. At the beginning and at the end of the experiment the participants filled extended surveys about: (1) dispositional (stable) personality traits (2) dispositional affective traits. These scores are considered as the dispositional factors of participants in our study. During the 30 work days, participants were asked to fill three *experience sampling* surveys about transient psychological states (personality and affect) that they have experienced in the last 30 minutes. The three daily reports were the same format as Big Five scales traditionally used for traits, with the exception that rather than describing themselves in general, participants described their personality-related behaviors during the previous 30 minutes (e.g. during the last 30 minutes, how well does talkative describe you?). It is very unlikely that people would have experienced significantly varying affect or personality states during such a short period of time. The surveys were triggered to be sent via email every working day at (11:00 AM, 2:00 PM and 5:00 PM). The participants were given 2.5 hours to fill the surveys. We refer to the first survey as the morning survey, the second survey as the midday survey and the third survey as the afternoon survey.

Table 1 summarizes the types of surveys used to capture different groups of states and traits. The Big Five Marker Scale (BFMS) is widely used to assess personality scores for extraversion, agreeableness, conscientiousness, emotional stability and creativity [56]. Therefore, BFMS was used in the Sociometric Badge Corpus at the beginning and at the end of the experiment to capture personality traits of participants [57]. Similarly, Multidimensional Personality Questionnaire (MPQ) was utilized to measure dispositional affective scores of participants [58].

**Table 1:** Surveys for personality and affect states and traits.

Group	Survey	Measurement
Personality	**States**	Extraversion
	Ten-Item Personality Inventory (TIPI)	Agreeableness
		Conscientiousness
	**Traits**	Emotional Stability
	Big Five Marker Scale (BFMS)	Creativity
Affect	**States**	High Positive Affect
	Positive and Negative Affect Schedule (PANAS)	High Negative Affect
	**Traits**	Low Positive Affect
	Multidimentional Personality Questionnaire (MPQ)	Low Negative Affect

On the other hand, experience sampling surveys elicit transient states of personality and affect (emotions) including questions about BIG5 personality scale and fifteen items concerning affective states. Questions in these surveys report participants’ states which were experienced in the last 30 minutes. For transient states of personality, the ten-item personality inventory TIPI was used in the experience sampling [59]. For each personality dimension e.g. extraversion, we recoded the reverse-scored items and then we computed the average of the two items (the standard item and the recoded reverse-scored item) that make up each dimension.

The short version of Positive and Negative Affect Schedule (PANAS) was used to evaluate the affective states of participants [60]. High positive affect (HPA) was assessed using 3 items: *enthusiastic*, *interested* and *active*. High negative affect (HNA) was assessed using 3 items: *sad*, *bored* and *sluggish*. Low positive affect was assessed using 2 items: *calm* and *relaxed* while low negative affect (LNA) was assessed using 2 items *lonely* and *isolated*.

The study lasted 30 working days and was performed during work hours at the premises of a research organization in northern Italy (for more details, see [57]). The data comprised 1,426 surveys by the 52 participant (see S1 Supporting Information for more details). The total number of Infrared hits detected by participants’ sensor while socializing with other participants is 114,642. When discussing the social interaction that a given participant was involved in, we refer to him/her as an *ego* and to the peers he/she interacted with as *alters*. Based on source, destination and time of those Infrared hits, we were able to construct the temporal face-to-face networks for each participant. When an ego’s Infrared sensor detects an alter, a directional transient edge is created between ego and alter and the weight of the edge is determined by the number of Infrared hits detected by ego’s sensor that are triggered by the recognition of alter’s sensor. The temporal networks consisted of 1,643 transient edges.

Personality and affect states’ scores are measured through continuous scales which are quantized into three ordinal levels—low, medium (neutral) and high—according to the three quantiles of their distributions in such a way that level L (Low) consisted of cases between the 0-th and the 33rd quantile; level N (Neutral) consisted of cases between the 33rd and the the 66th quantiles, and level H (High) consisted of cases above the latter (see S1 Supporting Information for more details). In [27], high and low state levels are considered as infectious while the medium (neutral) level is considered susceptible. In our case, state dynamics consist of changes (or lack thereof) of level between two consecutive surveys filled by a participant in a given day. Although our data is temporal, we focused on immediate single transitions within each state rather than looking at longer-term temporal trends. The addressed states fluctuate more than once on daily basis, so we just focused on the transitions that take place between two subsequent time periods and therefore we studied which social-situational factors are associated with those transitions.

Following the Italian regulations all participants were asked to sign an informed consent form and the study was conducted in accordance to them. The form and the general study was also approved by the Ethical Committee of Ca’ Foscari University of Venice.

Contact *intensity* is distinguished according to the level that the interacting partners were at the beginning of the relevant time interval (i.e., *t*) as illustrated in Fig 1(iii). The intensity of contacts with alters in a particular level (e.g. high) of the state is the ratio between the total number of infrared hits with those people and the total number of unique alters in the level. As a consequence, we have three different measures of situational factors: *L*, *N* and *H* corresponding to the intensity of contact with people in the low, medium (neutral) and high levels, respectively. Concerning individual dispositions, the level transitions for a given state were associated with the normalized score of the corresponding trait measured at the beginning of the study. In order to investigate the moderating role each trait plays in the association between transitions between levels of the state and the social-situational factors, we focused only on high and low trait scores (± 1 standard deviation).

### Statistical Models

For each possible transition between levels of a particular state (e.g. from Low to High in extraversion), our model consists of one dependent variable, the transition probability. Independent variables capture the corresponding trait score (*T*), the three situational measures *L*, *N* and *H* concerning contact intensity described above, the interaction effects T*L, T*N and T*H between trait and situational variables to account for the moderating effect of the former on the latter. The association between the dependent and the independent variables, including interactions, is modeled through logistic regression as shown in Eq 1. Following Banerjee et al [61], we used logistic regression instead of OLS regression (used by Hill et al [27]) because the value of the dependent variable is binary (0 if there is no transition and 1 otherwise). Let *X* → *Y* denote a transition by the ego from level *X* to level *Y* of some state *S* (*X* = *Y* denotes stability). Let *p*(*X* → *Y*) be the probability of this transition between two consecutive surveys. For each dynamic state *S*, we fit the following model:

$$\begin{array}{c} \begin{array}{c} ln\left(\frac{p(X\to Y)}{1-p(X\to Y}\right)=\alpha +\beta_{L}L+\beta_{N}N+\beta_{H}H+\beta_{T}T+\\ \beta_{T*L}T*L+\beta_{T*N}T*N+\beta_{T*H}T*H+\beta_{C}C \end{array} \end{array}$$ (1)

where *α* is a constant (intercept); *β*_*L*_, *β*_*N*_, *β*_*H*_ and *β*_*T*_ are coefficients of the *main* effects; *β*_T*L_, *β*_T*N_ and *β*_T*H_ are coefficients of the interaction effects between the trait *T* and the situational variables, and *β*_*C*_ is the coefficient of all the control variables. Our inclusion of individual-level trait effect reduces the likelihood that correlation is driven by choice of social connections, since it accounts for observable homophily [13, 61]. However, latent homophile effects cannot be completely ruled out [29].

The model also contains control variables, denoted *C*, whose role is modeled by parameter *β*_*C*_ (see S1 Supporting Information for more details). Importantly, it has been shown that the time of day can have a significant, universal (i.e. culture-independent) effect on mood –e.g. people tend to be more positive in the morning [62]. This *circadian rhythm* can be a major confounding variable in our analysis. By controlling for it, we eliminate a major confounding factor, since transitions between different levels of a particular state *S* (e.g. HPA) may be correlated due to spontaneous changes due to the time of day.

### Model and Parameter Estimation

Our dataset consists of repeated observations for each participant, so we expected to have correlations within observations of participants. Hence, we used generalized linear models to analyze our longitudinal data using unstructured covariance matrices whereby variances and covariances are estimated directly from the data. Generalized Estimation Equations (GEE) are used to estimate the parameters of our models. For each transition in each state, we used backward elimination that starts with a full model that contains all candidate variables. Then, we tested the effect of deletion of insignificant variables using QICC (Corrected Quasilikelihood under Independence Models Criterion) [63] iteratively until there is no further enhancement in the results. We evaluated the goodness of fit based on QICC which is an indicator of goodness of fit of models that use generalized estimating equations. Therefore, it can be utilized to choose between two models favoring the one with the smaller QICC. After we end up with the best sub-model for each state transition, we compare its QICC to the QICC of the null model thats contains only the intercept. If the best sub-model is better than the null model, then we retain it. Otherwise, we consider the null model.

Although it is not possible, with current statistical techniques, to check whether a decrease in QICC is statistically significant, the significance of the independent variables remain the same during the backward elimination in the majority of the cases. Moreover, backward elimination is widely used with information criterion for model selection [64]. Hence, the usage of QICC for model selection, as suggested by Pan [63], is capable of supporting the significance of our results.

## Results

We investigated 63 types of transitions by applying Eq 1 (3 transitions for each state level × three levels per state × 7 states). We analyzed 2,378 transitions and 3,390 stability cases in total (see Table 2 for other statistics).

**Table 2:** The maximum, minimum, median and mean number of transitions between levels of states for each transition.

**Transition**	**Max**	**Min**	**Median**	**Mean**
L → L	167	79	100	111
L → N	98	72	81	84
L → H	35	10	18	19
N → L	112	59	95	90
N → N	268	173	240	226
N → H	99	46	57	61
H → L	44	13	21	24
H → N	81	42	67	62
H → H	185	110	153	146

Here, for simplicity, we discuss the results of conscientiousness state only and we move the results of other states to the supporting information. Table 3 shows the detailed results of the conscientiousness state. The search for confirmation of the contagion model involves investigating the transition from the neutral level to another level, and transitions from the high or the low level back to the neutral level. According to Hill et al’s SISa model [27], a high or a low level is infectious only if (a) transitions to that level are only affected by the presence of people in that level, and (b) recovery is independent of social contact. We could not find any case that conformed to these conditions in conscientiousness state or the remaining six states as shown in S1 Supporting Information.

**Table 3:** Results of Conscientiousness State. The mere effects of social-situational factors (intensity with alters in each level: L, N and H) and corresponding traits of egos (T) are reported in the table, if they are statistically significant. The interaction results between the two effect are reported also (L*T, N*T and H*T), if they are statistically significant. The coefficients of the control variables are reported also: the main effect of the time of the day (period) and the interaction between the time of the day and the trait (period*T). Some reported coefficients are relatively small, therefore we used a threshold of 0.001 to consider them relevant. We focus more on the direction of the effect (increase or decrease in the probability) rather than the actual value of the effect.

**L to L**			**L to N**			**L to H**		
Variable	Coefficient	P-value	Variable	Coefficient	P-value	Variable	Coefficient	P-value
L	0.003	0.0017	Intercept	-0.441	0.0000	Intercept	-1.727	0.0000
H	-0.002	0.0129	N	-0.003	0.0000	(period = 1)	0.715	0.0130
N	0.003	0.0000				L	-0.034	0.0000
T	-0.269	0.0286				N	-0.009	0.0041
L*T	0.003	0.0127				T	0.751	0.1131
H*T	-0.002	0.0060				L*T	0.013	0.0000
						N*T	-0.008	0.0012
**N to L**			**N to N**			**N to H**		
Variable	Coefficient	P-value	Variable	Coefficient	P-value	Variable	Coefficient	P-value
Intercept	-0.408	0.0005	H	-0.001	0.0447	Intercept	-1.564	0.00
L	-0.0025	0.0231	T	-0.406	0.0005	(period = 1)	0.726	0.00
L*T	-0.006	0.0000	L*T	0.0025	0.0007	T	0.405	0.013
H*T	-0.002	0.0357	H*T	0.003	0.0010			
**H to L**			**H to N**			**H to H**		
Variable	Coefficient	P-value	Variable	Coefficient	P-value	Variable	Coefficient	P-value
Intercept	-1.579	0.0000	Intercept	-0.483	0.0002	N	0.003	0.0088
L	0.0024	0.0294	N	-0.0039	0.0004	T	0.5106	0.0000
H	-0.008	0.0255	T	-0.270	0.0050	N*T	-0.003	0.0144
L*T	-0.003	0.0006						

**Fig 2:**
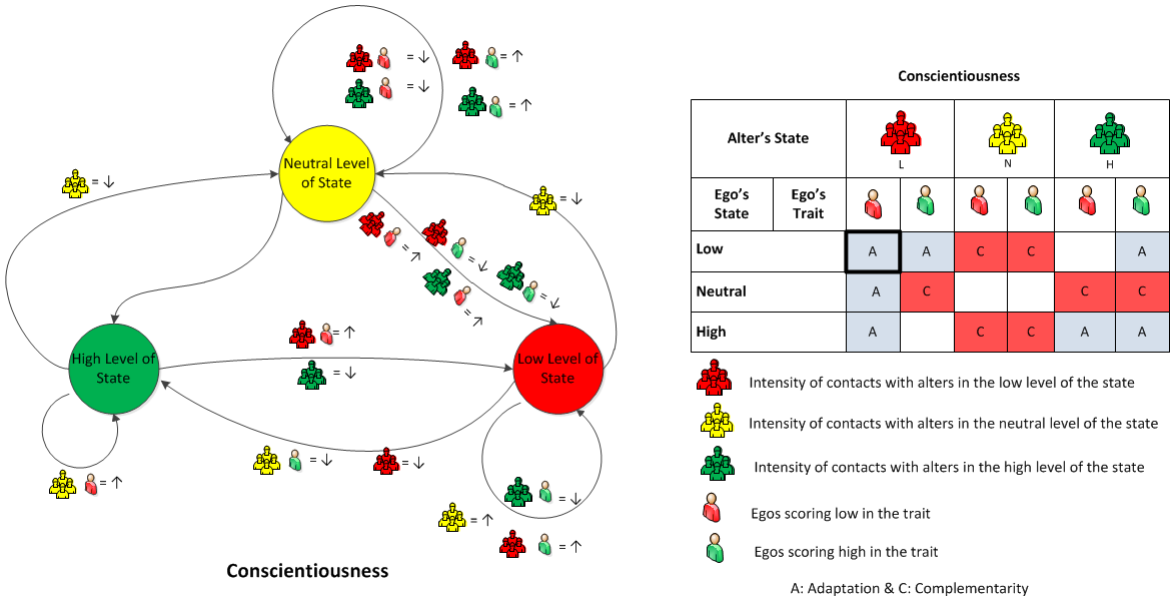
**(Left) Transition graph for conscientiousness:** Nodes represent conscientiousness level of the ego. Arrows represent transitions from one level to another. Transitions are labeled with conditions that affect the corresponding probabilities. Icons represent the conscientiousness levels of alters and ego’s trait level. Symbol ↑ (respectively ↓) indicates an increase (respectively decrease) in transition probability associated with the given combination of alters’ state level and ego’s trait level. For example, if the ego high in the conscientiousness state, then the probability of the ego transitioning to the high level decreases with ego’s contact with alters in the neutral level of the state. Another example is the transition from neutral to low level of conscientiousness, which is moderated by the ego’s trait score. If the ego is high in trait, then the probability of transition from a neutral state to a low state decreases with his contact with alters in high and low levels. But if the ego is low in the trait, then the probability increases instead. **(Right) Social influences:** The table summarizes the level transition graph by means of adaptation (A) and complementarity (C). Rows represent ego’s state levels; columns are labeled with alters’ state levels and sub-labeled with ego’s trait level (Low or High). Cells report the effects observed when egos in the corresponding state level and trait level interact with alters in the corresponding state level. For example, the square with thick border indicates that when the ego is low in conscientiousness state and also low in conscientiousness trait, contact with alters who are also low in conscientiousness state results in an adaptation effect. Empty cells lack statistically significant effects in a given combination.

We did, however, find evidence for *conditional contagion*. These are cases where the satisfaction of Hill et al’s definition was contingent on trait level. We found that transitions (*N* → *L*) do satisfy these requirements for people who have low scores in the conscientiousness trait. The same pattern was not observed for egos who have high scores in the conscientiousness trait; in this case, the probabilities of (*N* → *L*) decrease when the contact intensity with alters in state level *L* increases. This is illustrated in the state diagram in Fig 2 (left). No other cases conformed to the contagion model.

One reason for the failure of the contagion model is the fact that the role of alters in the neutral state level is not as passive as required by Hill et al [27] to define the recovery from infection. However, the presence of alters in the neutral level can be associated with changing probabilities of moving towards that level. This is what happens with alters in the neutral level whose presence was associated with a decrease in the probability of (*H* → *N*) and (*L* → *N*) transitions.

We observed also other roles of alters. For example, the presence of alters in the low level is associated with an increase in the probability of (*H* → *L*) for people who have low scores in the conscientiousness trait; while contact with alters in a low level of the state is associated with a decrease in the ego’s probability of transition (*N* → *N*).

Putting together all these observations, we introduce *attraction*, as a generalization of contagion. Intuitively, attraction obtains whenever increased interaction with alters in a state level different from the ego’s corresponds to either an increased probability for the ego to move towards that level or a decreased probability to move away from that level. Two things should be noted about this definition: (a) it does not require the “behavior” of the ego to fully conform to that of his/her alter, but simply to become more similar to it; (b) nothing is assumed concerning the recovery mechanism.

Attraction, by itself, is not enough, though. Often, we observed *repulsion*—cases in which increased interaction with people in a certain state level corresponds to decreasing probabilities of transition towards that level. This is what happens, for examples, with transitions (*L* → *N*) and (*H* → *N*). In the trait-conditional mode, it applies to the (*N* → *L*) transition of conscientiousness state for people who have high scores in the corresponding trait.

**Fig 3:**
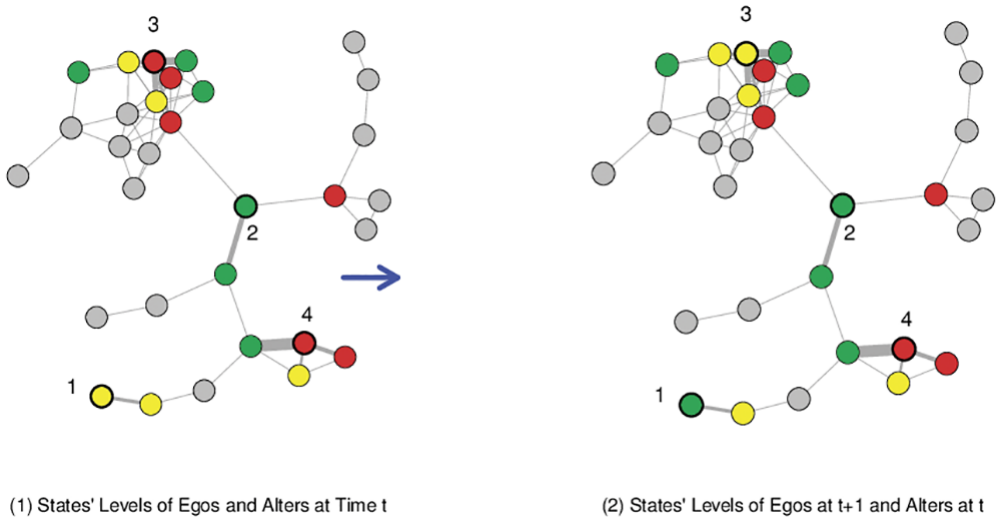
Two consecutive snapshots of 1st to 2nd survey in day 1, with the transient social network. Nodes are participants who filled both surveys. Edge thickness is proportional to contact intensity (IR hits) between surveys. Colors represent state levels (red:low, yellow:medium, green:high) and are shown only for egos 1–4 and their alters. The four types of social influence discussed in the text can be seen: ego 1 moved from the neutral level to the high level in the presence of interaction with an alter in the neutral level (push). Ego 2 remained in the high level in correspondence to intense contact with an alter in the high level (inertia). Ego 3 was in the low level and moved to neutral after intense contact with alters in the high level (attraction). Remarkably, the three egos have low scores in their corresponding traits. Ego 4 remained in the low level after contact with an alter in the high level (repulsion). The represented states are creativity for node 1, extraversion for nodes 2 and 4 and agreeableness for node 3.

Attraction and repulsion cover the associations between the intensity of contacts with alters in a level *different* from the ego’s initial one. But there is no reason not to expect that the intensity of contacts with people in the *same* level as the ego’s can also affect his/her transition probabilities. For instance, Ego 1 of Fig 3, who was in the neutral level and interacted with an alter in the same level, was *pushed* to the high level at the later sampling. Ego 2, who was in the high level and interacted with people in that same level, did not move, a situation we call *inertia*.

At the level of the whole sample, inertia is exemplified by increasing probability for people who are in the low level of the state but have high scores in the conscientiousness trait to remain at that level when they interact with alters in the low level of state (*L* → *L*). Similarly, the intensity of contacts with alters in the high level of the state is associated with a decrease in the probability of people in the same level to go to the low level (*H* → *L*). There are push effects in the conscientiousness state. However, there are some cases in other states (for push effects, see S1 Supporting Information).

In *attraction*, alters are in levels that are different from egos and therefore they encourage people to move to their levels; whereas, in *inertia*, alters are in the same levels of egos and encourage them to stay at the same levels. Both attraction and inertia represent a tendency of egos to *conform* to their alters. In *repulsion*, alters are in levels different from egos and therefore discourage people to move to their levels; whereas, in *push*, alters are in the same levels of egos and push people away from their levels. Therefore, repulsion and push represent the tendency to *diverge* from alters. We can, therefore, subsume our four effects under the *mimicry/adaptation* vs. *complementarity* distinction [42, 43, 50].

So far, we addressed one transition at a time. For a more complete picture, observe the state diagram of conscientiousness in Fig 2 (left) that summarizes the effect of the interplay between social-situational factors and individual dispositional factors on each transition. Due to space limitation, detailed results of other states can be found in S1 Supporting Information. We present our results in a more compact format in Fig 2 (right). Alters in the low level of the state attract egos to stay in the low level or switch to lower levels if those egos are already not conscientious by nature. Nevertheless, the alters cannot drag conscientious egos by nature towards their level unless the egos are already in the low level. Alters in the neutral level repulse egos to stay in their transient levels. Alters in the high level help egos to stay in the high level or move to their level except egos who are in the neutral level and have low trait scores.

## Discussion

### Summary and Implications

From a methodological perspective, our study shows how the combination of automated sensing of social interaction with high-frequency experience sampling [65] can build a detailed picture of the dynamics of personality and affect states in a sizable work community. This provides a significantly finer grained perspective compared to methodologies that exclusively rely on surveys and self-reported social interaction [21, 27]. This methodology can be applied well beyond the present study, e.g. to study the spread of healthy behavior or productive work practices in an organization.

Our methodology quantified complex patterns of social influence that go beyond the contagion metaphor [21, 27]. The associations we identified between social-situational aspects and transition probabilities—attraction, repulsion, inertia and push—account for a consistent majority (70%, 43 out of 63) of the transition types (3 transitions for each state level × three levels per state × 7 states) within the seven personality and affect states addressed in this work. They and their grouping under the headings of adaptation/mimicry and complementarity constitute an alternative and, we think, more appropriate taxonomy of social influence, one that is better suited to the ordinal nature of our psychological states. The model that emerges is one in which personality and affect states are not caught from someone else; they are not the result of mere contact, but from the ways egos (possibly unconsciously) respond to other people’s behaviors by either adapting to it—as discussed by social psychologists under the rubric of the “perception behavior link” or “chamaleon effect” [42]—or diverging from it. From this perspective, the ubiquitous moderating effect of individual differences (traits) corresponds to differential dispositions to respond to external solicitations.

Our observations suggest that interventions should not simply increase the prevalence of desired behavior. For example, while extrovertedly acting peers bring an introvert out of his shell (contagion), they push already extroverted persons towards introversion. So, simply adding extroverts to a group may not lead to an increase in overall extraversion (see S1 Supporting Information for similar generalizations).

In this work, we examined the role of corresponding traits in moderating the relationship between social-situational factors and variability within personality and affective states. Nevertheless, it was previously found that some traits such as extroversion and emotional stability traits are associated with fleeting affective states [66, 67]. In the future, it would be interesting to conduct similar investigations using all personality and affective traits.

### The Issue of Causality

Recently, the notion of contagion has been subject to considerable debate. In a seminal paper, Shalizi and Thomas identified situations in which latent homophily (similarity among interaction partners) together with causal effects from the homophilous trait cannot be distinguished, observationally, from contagion [29]. They argue that this raises barriers to many inferences social scientists would like to make about the underlying causal mechanisms. They further argue that these barriers can only be overcome by making exogenous assumptions about the causal architecture of the process in question.

While the contagion versus homophily debate is still ongoing [11, 30], it is important to clarify that our present paper is neutral on this particular issue. Rather, our results call for attention to a fundamental orthogonal issue: even if we take contagion as a given, it would still fail to account for the majority of potential social influence effects (i.e. effects that *may* be attributed to contact among individuals).

That is, regardless of whether true (causal) contagion exists, we show that all kinds of other associations (not just positive) can be observed when psychological dynamics are measured at higher resolution, and that these associations are moderated by individual stable traits. But the nature of our data still restricts us to correlational conclusions. For instance, just as contagion and homophily may be confounded in observational data [29], it may very well be the case that our observed ‘repulsion’ or ‘push,’ may be confounded with corresponding phenomena like ‘heterophily’ (the tendency to interact preferentially with people who exhibit opposing behavior or traits). Whether these associations reflect new causal mechanisms of influence remains an open question, and claims of causality must be based on well-justified assumptions [11]. The goal of this paper is precisely to motivate further investigation of these issues.

Said differently, we hope that our findings will encourage the broadening of the present research agenda on social dynamics [13, 27–31, 68, 69], from the specific idea of ‘contagion’ to the broader notion of ‘social influence’ which manifests itself through other psychological mechanisms (like mimicry/adaptation and complementarity). If true social influence exists, contagion is only a small part of it, and more complex interpersonal psychological dynamics are likely at play. Recognizing this is, we believe, a necessary prerequisite to unravelling the true nature of social dynamics.

## Supporting Information

S1 Supporting Information Contains the following. Sections: 1 Methods, 2 Results, 3 Detailed Results, 4 Broad Speculations and Further Work. Table A, Statistics of transitions between levels of each state. Table B, Surveys for personality an affect states and traits. Table C, Comparison of Goodness of Fit. Table D, Quantifying similarity between people according to their states. Table E, Extraversion Results. Table F, Agreeableness Results. Table G, Conscientiousness Results. Table H, Emotional Stability Results. Table I, Creativity Results. Table J, High Positive Affect (HPA) Results. Table K, Low Negative Affect (LNA) Results. Figure A, The composite social network of participants. Figure B, The degree distribution for each participant. Figure C, The interaction distribution for each participant. Figure D, Attraction and Repulsion. Figure E, Inertia and Push. Figure F, Social Influences. Figure G, Level transitions graph for extraversion stare. Figure H, Level transition graph for agreeableness state. Figure I, Level transition graph for conscientiousness state. Figure J, Level transition graph for emotional stability state. Figure K, Level transition graph for creativity state. Figure L, Level transition graph for high positive affect state. Figure M, Level transition graph for low negative affect state.
(PDF)

S1 Datasets Datasets of transitions in all personality and emotional states. (ZIP)

## References

[1] Asch SE. Opinions and social pressure Readings about the social animal. 1955;p. 17–26.

[2] Wood W. Attitude change: Persuasion and social influence. Annual review of psychology. 2000;51(1):539–570. 10.1146/annurev.psych.51.1.539 10751980

[3] Pentland A. Social Physics. Penguin Press; 2014.

[4] Cialdini RB, Goldstein NJ. Social influence: Compliance and conformity. Annu Rev Psychol. 2004;55:591–621. 10.1146/annurev.psych.55.090902.142015 14744228

[5] Fleeson W. Situation-based contingencies underlying trait-content manifestation in behavior. Journal of Personality. 2007;75(4):825–861. 10.1111/j.1467-6494.2007.00458.x 17576360

[6] Bond RM, Fariss CJ, Jones JJ, Kramer AD, Marlow C, Settle JE, et al A 61-million-person experiment in social influence and political mobilization. Nature. 2012;489(7415):295–298. 10.1038/nature11421 22972300 PMC3834737

[7] Mani A, Rahwan I, Pentland A. Inducing Peer Pressure to Promote Cooperation. Scientific reports. 2013;3(1735):1–9. 10.1038/srep01735 PMC3636514 23619166

[8] Schultz PW, Nolan JM, Cialdini RB, Goldstein NJ, Griskevicius V. The constructive, destructive, and reconstructive power of social norms. Psychological science. 2007;18(5):429–434. 10.1111/j.1467-9280.2007.01917.x 17576283

[9] Burt RS. Social contagion and innovation: Cohesion versus structural equivalence. American journal of Sociology. 1987;92(6):1287–1335. 10.1086/228667

[10] Coleman J, Katz E, Menzel H. The diffusion of an innovation among physicians Sociometry. 1957;p. 253–270.

[11] Christakis NA, Fowler JH. Social contagion theory: examining dynamic social networks and human behavior. Statistics in Medicine. 2013;(32):556–577. 10.1002/sim.5408 22711416 PMC3830455

[12] Christakis N, Fowler J. The Spread of Obesity in a Large Social Network over 32 Years. New England Journal of Medicine. 2007;357(4):370–379. 10.1056/NEJMsa066082 17652652

[13] Coviello L, Sohn Y, Kramer AD, Marlow C, Franceschetti M, Christakis NA, et al Detecting Emotional Contagion in Massive Social Networks. PLOS ONE. 2014;9(3):e90315 10.1371/journal.pone.0090315 24621792 PMC3951248

[14] Kramer AD, Guillory JE, Hancock JT. Experimental evidence of massive-scale emotional contagion through social networks. Proceedings of the National Academy of Sciences. 2014;111(24):8788–8790. 10.1073/pnas.1320040111 PMC4066473 24889601

[15] Madan A, Moturu ST, Lazer D, Pentland AS. Social Sensing: Obesity, Unhealthy Eating and Exercise in Face-to-Face Networks In: Wireless Health 2010. New York, NY, USA: ACM; 2010 p. 104–110.

[16] Fowler JH, Christakis NA. Cooperative Behavior Cascades in Human Social Networks. PNAS: Proceedings of the National Academy of Sciences. 2010;107(12):5334–5338. 10.1073/pnas.0913149107 PMC2851803 20212120

[17] Jordan JJ, Rand DG, Arbesman S, Fowler JH, Christakis NA. Contagion of cooperation in static and fluid social networks. PLOS ONE. 2013;8(6):e66199 10.1371/journal.pone.0066199 23840422 PMC3686805

[18] Suri S, Watts DJ. Cooperation and contagion in web-based, networked public goods experiments. PLOS ONE. 2011;6(3):e16836 10.1371/journal.pone.0016836 21412431 PMC3055889

[19] Tsvetkova M, Macy MW. The Social Contagion of Generosity. PLOS ONE. 2014;9(2):e87275 10.1371/journal.pone.0087275 24551053 PMC3923723

[20] Christakis N, Fowler J. The Collective Dynamics of Smoking in a Large Social Network. New England Journal of Medicine. 2008;358(21):2249–2258. 10.1056/NEJMsa0706154 18499567 PMC2822344

[21] Fowler J, Christakis N. Dynamic Spread of Happiness in a Large Social Network: Longitudinal Analysis over 20 years in the Framingham Heart Study. British Medical Journal. 2008;337(a2338):1–9. 10.1136/bmj.a2338 PMC2600606 19056788

[22] Pugh SD. Service with a smile: emotional contagion in the service encounter. The Academy of Management Journal. 2001;44(5):1018–1027. 10.2307/3069445

[23] Madan A, Cebrian M, Lazer D, Pentland A. Social sensing for epidemiological behavior change In: Proceedings of the 12th ACM international conference on Ubiquitous computing. ACM; 2010 p. 291–300.

[24] Howes MJ, Hokanson JE, Loewenstein DA. Induction of depressive affect after prolonged exposure to a mildly depressed individual. Journal of Personality and Social Psychology. 1985;49(4):1110–1113. 10.1037/0022-3514.49.4.1110 4057047

[25] Hatfield E, Cacioppo J, Rapson RL. Emotional contagion. Cambridge University Press; 1994.

[26] Barsade SG. The Ripple Effect: Emotional Contagion and its Influence on Group Behavior. Administrative Science Quarterly. 2002;47(4):644–675. 10.2307/3094912

[27] Hill AL, Rand DG, Nowak MA, Christakis N. Emotions as infectious diseases in a large social network: the SISa model. Proceedings of Royal Society B: Biological Sciences. 2010;277(1701):3827–3835. 10.1098/rspb.2010.1217 PMC2992714 20610424

[28] Hill AL, Rand DG, Nowak MA, Christakis NA. Infectious disease modeling of social contagion in networks. PLOS computational biology. 2010;6(11):e1000968 10.1371/journal.pcbi.1000968 21079667 PMC2973808

[29] Shalizi CR, Thomas AC. Homophily and contagion are generically confounded in observational social network studies. Sociological Methods & Research. 2011;40(2):211–239. 10.1177/0049124111404820 22523436 PMC3328971

[30] Steeg GV, Galstyan A. Statistical Tests for Contagion in Observational Social Network Studies. In: Paper presented at International Conference on Artificial Intelligence and Statistics; 2013.

[31] Aral S, Muchnik L, Sundararajan A. Distinguishing influence-based contagion from homophily-driven diffusion in dynamic networks. Proceedings of the National Academy of Sciences. 2009;106(51):21544–21549. 10.1073/pnas.0908800106 PMC2799846 20007780

[32] Dodds PS, Watts DJ. A generalized model of social and biological contagion. Journal of Theoretical Biology. 2005;232(4):587–604. 10.1016/j.jtbi.2004.09.006 15588638

[33] Friedkin NE. The attitude-behavior linkage in behavioral cascades. Social Psychology Quarterly. 2010;73(2):196–213.

[34] Smilkov D, Hidalgo CA, Kocarev L. Beyond network structure: How heterogeneous susceptibility modulates the spread of epidemics. Scientific reports. 2014;4:1–7. 10.1038/srep04795 PMC3999455 24762621

[35] Zelenski JM, Larsen RJ. The distribution of basic emotions in everyday life: A state and trait perspective from experience sampling data. Journal of Research in Personality. 2000;34(2):178–197. 10.1006/jrpe.1999.2275

[36] Fleeson W. Moving personality beyond the person-situation debate the challenge and the opportunity of within-person variability. Current Directions in Psychological Science. 2004;13(2):83–87. 10.1111/j.0963-7214.2004.00280.x

[37] Fleeson W, Noftle E. The end of the person-situation debate: an emerging synthesis in the answer to the consistency question. Social and Personality Psychology Compass. 2008;2:1667–1684. 10.1111/j.1751-9004.2008.00122.x

[38] Costa PT, McCrae RR. The revised neo personality inventory (neo-pi-r). The SAGE handbook of personality theory and assessment. 2008;2:179–198.

[39] Fleeson W. Toward a structure- and process-integrated view of personality: traits as density distribution of states. Journal of Personality and Social Psychology. 2001;80(6):1011–1027. 10.1037/0022-3514.80.6.1011 11414368

[40] Friedkin NE, Johnsen EC. Social influence network theory: a sociological examination of small group dynamics. vol. 33 Cambridge University Press; 2011.

[41] Watson D, Clark LA, Tellegen A. Development and validation of brief measures of positive and negative affect: the PANAS scales. Journal of Personality and Social Psychology. 1988 6;54(6):1063–1070. Available from: http://view.ncbi.nlm.nih.gov/pubmed/3397865. 10.1037/0022-3514.54.6.1063 3397865

[42] Chartrand TL, Bargh JA. The chameleon effect: The perception–behavior link and social interaction. Journal of personality and social psychology. 1999;76(6):893 10.1037/0022-3514.76.6.893 10402679

[43] Tiedens LZ, Fragale AR. Power moves: complementarity in dominant and submissive nonverbal behavior. Journal of personality and social psychology. 2003;84(3):558 10.1037/0022-3514.84.3.558 12635916

[44] Pentland A. The New Science of Building Great Teams. Harvard Business Review. 2012;90(4):60–70.23074865

[45] Barrick MR, Mount MK, Judge TA. Personality and Performance at the Beginning of the New Millennium: What Do We Know and Where Do We Go Next? International Journal of Selection and Assessment. 2001;9(1&2):9–30. 10.1111/1468-2389.00160

[46] Ilies R, Judge TA. Understanding the dynamic relationships among personality, mood, and job satisfaction: A field experience sampling study. Organizational Behavior and Human Decision Processes. 2002;89(2):1119–1139. 10.1016/S0749-5978(02)00018-3

[47] Bell ST. Deep-level composition variables as predictors of team performance: a meta-analysis. Journal of Applied Psychology. 2007;92(3):595–615. 10.1037/0021-9010.92.3.595 17484544

[48] Barsade S, Brief AP, Spataro SE. The affective revolution in organizational behavior: The emergence of a paradigm Organizational Behavior: The State of the Science. 2003;p. 3–52.

[49] Olguín Olguín D, Waber BN, Kim T, Mohan A, Ara K, Pentland A. Sensible organizations: Technology and methodology for automatically measuring organizational behavior. IEEE Transactions on Systems, Man, and Cybernetics, Part B (Cybernetics). 2009;39(1):43–55. 10.1109/TSMCB.2008.2006638 19150759

[50] Pentland AS. Honest signals. MIT press; 2010.

[51] Cattuto C, Van den Broeck W, Barrat A, Colizza V, Pinton JF, Vespignani A. Dynamics of person-to-person interactions from distributed RFID sensor networks. PLOS ONE. 2010;5 10.1371/journal.pone.0011596 20657651 PMC2904704

[52] Salathé M, Kazandjieva M, Lee JW, Levis P, Feldman MW, Jones JH. A high-resolution human contact network for infectious disease transmission. Proceedings of the National Academy of Sciences. 2010;107(51):22020–22025. 10.1073/pnas.1009094108 PMC3009790 21149721

[53] Stehlé J, Voirin N, Barrat A, Cattuto C, Isella L, Pinton JF, et al High-resolution measurements of face-to-face contact patterns in a primary school. PLOS ONE. 2011;6(8):e23176 10.1371/journal.pone.0023176 21858018 PMC3156713

[54] Todd PM, Penke L, Fasolo B, Lenton AP. Different cognitive processes underlie human mate choices and mate preferences. Proceedings of the National Academy of Sciences. 2007;104(38):15011–15016. 10.1073/pnas.0705290104 PMC1986604 17827279

[55] Csikszentmihalyi M, Hunter J. Happiness in everyday life: The uses of experience sampling. Journal of Happiness Studies. 2003;4(2):185–199. 10.1023/A:1024409732742

[56] Perugini M, L B. The Big Five Marker Scales (BFMS) and the Italian AB5C taxonomy: Analyses from an emic-etic perspective; 2012.

[57] Lepri B, Staiano J, Rigato G, Kalimeri K, Finnerty A, Pianesi F, et al The sociometric badges corpus: A multilevel behavioral dataset for social behavior in complex organizations In: Privacy, Security, Risk and Trust (PASSAT), International Confernece on Social Computing (SocialCom). IEEE; 2012 p. 623–628.

[58] Tellegen A, Waller NG. Exploring Personality Through Test Construction: Development of the Multidimensional Personality Questionnaire In: Boyle GJ, Matthews G, Saklofske DH, editors. The SAGE Handbook of Personality Theory and Assessment: Volume 2—Personality Measurement and Testing. SAGE Publications Ltd; 2008 p. 261–293.

[59] Gosling S, Rentfrow P, Swann W. A very brief measure of the Big-Five personality domains. Journal of Research in Personality. 2003;37(6):504–528. 10.1016/S0092-6566(03)00046-1

[60] Watson D, Clark LA, Tellegen A. Development and validation of brief measures of positive and negative affect: the PANAS scales. Journal of personality and social psychology. 1988;54(6):1063 10.1037/0022-3514.54.6.1063 3397865

[61] Banerjee A, Chandrasekhar AG, Duflo E, Jackson MO. The diffusion of microfinance. Science. 2013;341 (6144). 10.1126/science.1236498 23888042

[62] Golder SA, Macy MW. Diurnal and seasonal mood vary with work, sleep, and daylength across diverse cultures. Science. 2011;333(6051):1878–1881. 10.1126/science.1202775 21960633

[63] Pan W. Akaike’s information criterion in generalized estimating equations. Biometrics. 2001;57(1):120–125. 10.1111/j.0006-341X.2001.00120.x 11252586

[64] Royston P, Moons KG, Altman DG, Vergouwe Y. Prognosis and prognostic research: developing a prognostic model. Bmj. 2009;338:b604 10.1136/bmj.b604 19336487

[65] Killingsworth MA, Gilbert DT. A wandering mind is an unhappy mind. Science. 2010;330(6006):932–932. 10.1126/science.1192439 21071660

[66] Tellegen A. Structures of mood and personality and their relevance to assessing anxiety, with an emphasis on self-report. 1985;.

[67] Watson D, Clark LA. Negative affectivity: the disposition to experience aversive emotional states. Psychological bulletin. 1984;96(3):465 10.1037/0033-2909.96.3.465 6393179

[68] Aral S, Walker D. Identifying influential and susceptible members of social networks. Science. 2012;337(6092):337–341. 10.1126/science.1215842 22722253

[69] Muchnik L, Aral S, Taylor SJ. Social influence bias: A randomized experiment. Science. 2013;341(6146):647–651. 10.1126/science.1240466 23929980

