# Supplementary material for: Beyond Contagion: Reality Mining Reveals Complex Patterns of Social Influence
Source: PLoS One. 2015 Aug 27;10(8):e0135740. doi: 10.1371/journal.pone.0135740 (PMC4551670; doi:10.1371/journal.pone.0135740)
Supplement: S1 Supporting Information — Sections: 1 Methods, 2 Results, 3 Detailed Results, 4 Broad Speculations and Further Work. Table A, Statistics of transitions between levels of each state. Table B, Surveys for personality an affect states and traits. Table C, Comparison of Goodness of Fit. Table D, Quantifying similarity between people according to their states. Table E, Extraversion Results. Table F, Agreeableness Results. Table G, Conscientiousness Results. Table H, Emotional Stability Results. Table I, Creativity Results. Table J, High Positive Affect (HPA) Results. Table K, Low Negative Affect (LNA) Results. Figure A, The composite social network of participants. Figure B, The degree distribution for each participant. Figure C, The interaction distribution for each participant. Figure D, Attraction and Repulsion. Figure E, Inertia and Push. Figure F, Social Influences. Figure G, Level transitions graph for extraversion stare. Figure H, Level transition graph for agreeableness state. Figure I, Level transition graph for conscientiousness state. Figure J, Level transition graph for emotional stability state. Figure K, Level transition graph for creativity state. Figure L, Level transition graph for high positive affect state. Figure M, Level transition graph for low negative affect state. (PDF) [file pone.0135740.s001.pdf]

Supporting Information for: Beyond contagion: Reality mining reveals complex patterns of social influence

Aamena Alshamsi[1], Fabio Pianesi[2], Bruno Lepri[2,3], Alex Pentland[3], and Iyad Rahwan[1,4,†]

[1]*Masdar Institute of Science & Technology, Abu Dhabi 54224, UAE*
[2]*Foundation Bruno Kessler, Trento, Italy*
[3]*Media Laboratory, Massachusetts Institute of Technology, Cambridge, MA*
[4]*School of Informatics, University of Edinburgh, Edinburgh EH8 9AB, UK*
[†]*Correspondence should be addressed to* `irahwan@acm.org`

1 Methods

1.1 Data Collection

Through an experiment, the SocioMetric Badges Corpus tracked the activities of 52 participants [6]. The fifty two participants are employees in a research institution in Italy who volunteered to participate in the experiment for six weeks (working days are considered only). They belong to five units whereby all the employees of these units participated in the experiment along with the heads of these units. Their ages range from 23 to 53 with an average of 36. Forty seven participants are men (90.3%) and five are women (9%). Forty four participants are Italian (84%) and eight participants are from other countries (15.3%). Forty-six out of the fifty-three participants were researchers in computer science belonging to four research groups; the remaining six participants were part of the full-time IT support staff.

1.2 Procedure

At the beginning and at the end of the experiment, the participants filled extended surveys about: (1) dispositional (stable) personality traits (2) dispositional affective traits. These scores are considered as the dispositional factors of participants in our study. During the 6 weeks, participants were asked to fill three *experience sampling* surveys about transient psychological states (personality and affect) that they have experienced in the last 30 minutes. It is very unlikely that people would have experienced significantly varying affect or personality states during such a short period of time. The surveys were triggered to be sent via email every working day at (11:00 AM, 2:00 PM and 5:00 PM). The participants were given 2.5 hours to fill the surveys. We refer to the first survey

as the morning survey, the second survey as midday survey and the third survey as the afternoon survey.

Table B summarizes the types of surveys used to capture different groups of states and traits. The Big Five Marker Scale (BFMS) is widely used to assess personality scores for extraversion, agreeableness, conscientiousness, emotional stability and creativity [9]. Therefore, BFMS was used in the Sociometric Badge Corpus at the beginning and at the end of the experiment to capture personality traits of participants [6]. Similarly, Multidimensional Personality Questionnaire (MPQ) was utilized to measure dispositional affective scores of participants [10].

On the other hand, experience sampling surveys elicit transient states of personality and affect (emotions) including questions about BIG5 personality scale and fifteen items concerning affective states. Questions in these surveys report participants' states which were experienced in the last 30 minutes. For transient states of personality, the ten-item personality inventory TIPI was used in the experience sampling [4]. For each personality dimension e.g. extraversion, we recoded the reverse-scored items and then we computed the average of the two items (the standard item and the recoded reverse-scored item) that make up each dimension.

The short version of Positive and Negative Affect Schedule (PANAS) was used to evaluate the affective states of participants [11]. High positive affect (HPA) was assessed using 3 items: *enthusiastic*, *interested* and *active*. High negative affect (HNA) was assessed using 3 items: *sad*, *bored* and *sluggish*. Low positive affect was assessed using 2 items: *calm* and *relaxed* while low negative affect (LNA) was assessed using 2 items *lonely* and *isolated*.

After we calculate the scores of personality and affective states of each participant in each filled survey, we centered all the scores of personality and affect dynamic states using the median of each state. We generated the quantiles for each state, discretizing the scores of personality and affect dynamic states into three ordered levels (Low, Neutral and High).The three levels were identified on the basis of the 33rd and 66th quantiles of the state scores distribution in such a way that level L (low) consisted of cases between the 0-th and the 33rd quantile; level N (neutral) consisted of cases between the 33rd and the the 66th quantiles, and level H (high) consisted of cases above the latter.

Note that the experience sampling method has a long history and is highly reliable in measuring dynamics of psychological states within individuals [1]. For those interested in the caveats around the use of experience sampling, we also point to extensive discussions elsewhere [2].

The participants wore SocioMetric Badges every working day within the institution. These sensors are equipped with accelerometers, audio, Bluetooth and Infrared to respectively capture: body movements, prosodic speech features, co-location with other individuals and face-to-face interactions [8]. We harnessed Infrared (IR) transmissions to detect face-to-face interactions between people. In order for a badge to be detected through IR, two of them must have a direct line of sight and the receiving badge's IR must be within the transmitting badge's IR signal cone of height $h <= 1$ meter and a radius of $r <= htan\theta$, where θ = $15°$ degrees; the infrared transmission rate (TRir) was set to 1Hz.

1.3 Preprocessing the surveys

The data comprises 1,426 surveys by the 52 participants. Ideally, the number of filled surveys should be 4,680 (52 participants $\times$ 3 daily surveys $\times$ 30 working days). However, participants reported absence from work 536 times [7]. This reduces the number of expected responses to 4,144. We addressed transitions in states levels between daily first surveys and second surveys and transitions in states levels between the second and the third surveys. Therefore, the number of daily expected transitions is 2. This further reduces the number of records (transitions) by 1,560 (52 participants $\times$ 30 days) to reach 2,582 expected records. Also, it has been observed that the majority of participants used to leave the organization before 5PM on Fridays afternoon. This further reduces the number of expected responses. The response rate of filling surveys is 83.9% according to Lepri et al [7] which means that participants skip some surveys despite their availability at work. Unfilling surveys has an impact on the number of transitions. For example, if a participant missed the second daily survey, then two transitions will be missing: transition from first to second survey and transition from second survey. Also, we did not consider the transitions of egos in which the alters have not filled the corresponding surveys. Therefore, we ended up having only 1,426 surveys.

Transient states of personality and affect First we centered all the scores of personality and affect dynamic states using the median of each state. Then, we generated the quantiles for each state, discretized the scores of personality and affect dynamic states into three ordered levels (Low, Neutral and High).The three levels were identified on the basis of the 33rd and 66th quantiles of the state scores distribution in such a way that level L (low) consisted of cases between the 0-th and the 33rd quantile; level N (neutral) consisted of cases between the 33rd and the the 66th quantiles, and level H (high) consisted of cases above the latter. We discarded both high negative affect and low positive affect because their distributions are very skewed making it difficult to identify appropriate intervals for each level of these states. Hence, high negative affect and low positive affect were not discussed in the results.

Accordingly, the level of each participant in each dynamic state recorded at a given survey is identified to be one of the three above levels. We leveraged those levels to extract the transitions in levels of dynamic states between two consecutive survey in the same day. Also, we used those levels to extract the social-situational factors by categorizing the social ties (other participants) whom the Infrared sensor of a particular participant detected between two consecutive surveys to be one of the three predefined levels. Remarkably, we used the lagged levels of the social ties. For example, the transition in question is taking place between the morning survey (time t) and midday survey (time $t + 1$) for a particular participant. Therefore, we consider the scores of social ties recorded in the morning survey (time t). The statistics of transitions in each level is provided in Table A.

Dispositional Traits of Personality and Affect We considered the trait scores that were reported by participants at the beginning of the experiment. Then, we normalized the trait scores of participants using the mean and the standard deviation. To discuss the statistical interaction between traits and social-situational factors associated with a given transition, we focused on only on participants

Transition	Extraversion	Agreeableness	Conscientiousness	Emotional Stability	Creativity	HPA	LNA
L → L	79	100	167	147	107	91	89
L → N	77	98	83	72	97	81	79
L → H	35	25	21	10	14	18	13
N → L	59	90	112	79	102	95	95
N → N	240	254	173	187	246	217	268
N → H	99	51	47	57	60	65	46
H → L	44	25	18	20	21	13	25
H → N	76	71	50	67	45	81	42
H → H	115	110	153	185	132	163	167

Transition to	Extraversion	Agreeableness	Conscientiousness	Emotional Stability	Creativity	HPA	LNA
L	22.087	26.092	36.044	29.854	27.913	24.150	25.364
N	52.752	58.425	46.575	48.154	54.114	51.705	52.925
H	37.275	29.712	38.502	41.653	33.226	37.730	34.451

Table A: Statistics of transitions between levels of each state: The first sub-table presents the number of cases per transition in each state. The second sub-table presents the percentage of transitions to each target level of each state.

with high scores in the trait (+1 standard deviation) and participants with low scores in the trait (-1 standard deviation). By using this method, we are able to know how levels of traits moderates the association between the social-situational factors and transitions in states. For example, we are interested to know how introverts respond to an increase in the intensity of contacts with others in a certain extraversion level in comparison to extroverts' response to the same increase.

One might argue that there might be a systematic difference in how emotive marketing and sales people are compared to engineers. However, our control for personality traits would capture these variations. In fact, our control for traits goes significantly beyond broad classifications based on profession, since it captures underlying personality types using a systematic approach founded in well-established dimensions of personality.

Group	Survey	Measurement
Personality	**States**	Extraversion
	Ten-Item Personality Inventory (TIPI)	Agreeableness
		Conscientiousness
	**Traits**	Emotional Stability
	Big Five Marker Scale (BFMS)	Creativity
Affect	**States**	High Positive Affect
	Positive and Negative Affect Schedule (PANAS)	High Negative Affect
	**Traits**	Low Positive Affect
	Multidimentional Personality Questionnaire (MPQ)	Low Negative Affect

Table B: Surveys for personality and affect states and traits.

1.4 Dynamic Social networks

We created dynamic temporal networks of face-to-face interaction for each participant . Between each two subsequent surveys a participant filled, we created the participant's temporal social network based on the social ties that the Infrared sensor had detected. We considered only social networks that were formed between morning and midday daily surveys or between midday and afternoon daily surveys. Based on this, we considered the transition in personality and emotional states of egos between these surveys (time t and time $t+1$) and compared them to lagged states' levels of alters and traits of egos at time $t+1$. To generate our social networks, we exploited the Sociometric Badges Corpus, first introduced by Lepri et al. [6].

We harnessed the experience sampling data and Infrared readings to create the dynamic social networks for participants. The time boundary of each social network is delineated by the time of subsequent surveys a participant filled. Between the two surveys, all face-to-face interactions detected via Infrared sensors are considered to be the social-situational factors for the participant. Particularly, what matters is the level at which each alter was at time t (morning survey if the transition was initiated at the morning survey and midday surveys if the transition was initiated at midday survey).

1.5 Model and Parameter Estimation

Our dataset consists of repeated observations for each participant, so we expected to have correlations within observations of participants. Hence, we used generalized linear models to analyze our longitudinal data using unstructured covariance matrices whereby variances and covariances are estimated directly from the data. Generalized Estimation Equations (GEE) are used to estimate the parameters of our models. For each transition in each state, we used backward elimination that starts with a full model that contains all candidate variables. Then, we tested the effect of deletion of insignificant variables using QICC (Corrected Quasilikelihood under Independence Models Criterion) iteratively until there is no further enhancement in the results. We evaluated the goodness of fit based on QICC which is an indicator of goodness of fit of models that use generalized estimating equations. Therefore, it can be utilized to choose between two models favoring the one with the smaller QICC. After we end up with the best sub-model for each state transition, we compare its QICC to the QICC of the null model thats contains only the intercept. If the best sub-model is better than the null model, then we retain it. Otherwise, we consider the null model. Table C compares between the QICC of our best sub-model and the null model for each transition in each state. If the QICC of the null model is better, then we report only the QICC of the null model.

In other words, we used QICC to penalize having complex models that might cause overfitting. Therefore, for each transition in each state, we start with having the complete model $Model1$ that includes all of the candidate independent variables and calculate the QICC of the model. Then, each time we encounter a statistically insignificant independent variable, we drop the variable, run the model again without this variable and calculate the QICC of the reduced model $Model2$. If the QICC of $Model2$ is higher than the QICC of $Model1$, we consider the results of Model1 and report them in the paper. If there is any statistically insignificant variable, we drop the variable from the model, run the model again without this variable $Model3$ and calculate the QICC of

$Model3$. Then, we compare QICC of $Model2$ and QICC of $Model3$. If the QICC of $Model2$ is lower, then we report the results of $Model2$. Otherwise, we repeat the same process until there is no further reduction in QICC.

Although we do not have any statistical test to check whether the decrease in QICC is statistically significant, the significance of the independent variables remain the same during the backward elimination in the majority of the cases. Hence, we believe that using QICC for model selection is capable of supporting the significance of our results.

Extraversion

Transition	Our Model	Null Model
L to L	258.823	261.053
L to N		260.987
L to H	193.796	194.776
N to L	346.415	348.420
N to N	536.751	540.690
N to H		460.236
H to L	231.949	239.164
H to N		303.378
H to H	321.325	329.069

Agreeableness

Transition	Our Model	Null Model
L to L	302.3	309.7
L to N	303	308.9
L to H	167.87	171.7
N to L		435.9
N to N	519.7	520.5
N to H	314.4	316.1
H to L		171.5
H to N	262.045	270.8
H to H	271.5	290.359

Conscientiousness

Transition	Our Model	Null Model
L to L	355.184	367.347
L to N	336.434	340.500
L to H	156.765	167.498
N to L	420.844	430.617
N to N	455.696	463.190
N to H	273.47	286.849
H to L	140.832	142.188
H to N	236.687	247.471
H to H	263.305	281.446

Emotional Stability

Transition	Our Model	Null Model
L to L		305.981
L to N		293.489
L to H	91.054	101.587
N to L	359.896	367.961
N to N	434.818	442.637
N to H	305.636	310.707
H to L	161.530	163.776
H to N		315.597
H to H		354.350

Creativity

Transition	Our Model	Null Model
L to L	299.310	304.301
L to N		302.174
L to H	111.789	126.067
N to L		463.480
N to N		551.166
N to H	348.1	356.400
H to L		153.455
H to N	205.969	226.689
H to H	251.582	270.706

High Positive Affect (HPA)

Transition	Our Model	Null Model
L to L	400.737	406.689
L to N		310.175
L to H	294.914	306.805
N to L	196.9	206.045
N to N	175.454	176.941
N to H		203.480
H to L	355.656	374.595
H to N	307.305	316.248
H to H	443.738	481.045

Low Negative Affect (LNA)

Transition	Our Model	Null Model
L to L	251.309	252.927
L to N		250.422
L to H	107.607	111.148
N to L	449.815	452.999
N to N	529.541	531.894
N to H	302.882	308.433
H to L	169.170	184.558
H to N	232.818	242.474
H to H	263.323	302.914

Table C: Comparison of Goodness of Fit: We compare between the QICC of our best sub-model and the QICC of the null model of each transition in each state. Only the QICC of the null model is reported if it is less (better) than the QICC of the sub-model.

For each possible transition between levels of a particular state, our model consists of: one dependent variable, the transition probability; nine independent variables that capture the corresponding trait score (T) and the three situational measures concerning contact intensity described above: L, N and H. The model also contains the interaction effects between trait and situational

variables, in order to account for the moderating effect of the former on the latter: $T * L$, $T * N$ and $T * H$. The level transitions might take place spontaneously due to the time of the day as highlighted by Golder and Macy [3]. Therefore, we added a time period variable that can act as a control variable to capture possible diurnal rhythms P. Particularly, we are interested to study the effect of time of day at time $t + 1$ for the transition that takes place between time t and time $t + 1$. Therefore, the time period could be either midday or afternoon. Also, we expect to have an interaction between the time of the day and the corresponding trait. Therefore, we added one more control variable $P * T$. The association between the dependent and the independent variables, including interactions, is modeled through logistic regression as shown in Equation 1. We used logistic regression instead of OLS regression (used by Hill et al [5]) because the value of the dependent variable is binary (0 if there is no transition and 1 otherwise). Let $X \to Y$ denotes a transition by the ego from level X to level Y of some state S (we permit $X = Y$ denoting stability). Let $p(X \to Y)$ be the probability of this transition between two consecutive surveys. For a given dynamic personality or affect state *S*:

$$
\begin{aligned}
ln(\frac{p(X \to Y)}{1 - p(X \to Y}) = \alpha + \beta_L L + \beta_N N + \beta_H H + \beta_T T + \\
\beta_{T*L} T * L + \beta_{T*N} T * N + \beta_{T*H} T * H + \beta_P P + \beta_{P*T} P * T
\end{aligned}
\tag{1}
$$

where α is a constant (intercept); T, L, N and H are as explained above. β_L, β_N, β_H and β_T are the coefficients of the *main* effects L, N, H and T, respectively. β_{T*L}, β_{T*N} and β_{T*H} are the coefficients of the interaction effects between the trait T and the situational variables L, N and H, respectively. β_P is the coefficient of time of the day and β_{P*T} is the coefficient of the interaction between the corresponding trait and the time of the day.

2 Results

First, we discuss some descriptive statistics about network dynamics and potential homophily in the networks of participants. Second, we describe the four social influence processes that we identified in our model. Third, we describe our results in the context of these processes.

2.1 Descriptive Statistics

We provide some descriptive statistics about social network dynamics. Fig. B and Fig. C show the degree distribution and interaction distribution. In most cases, the dynamic networks of participants vary in terms of the number of alters and the total number of interactions.

Also, we quantify the similarity between egos and alters in terms of their state levels. We calculated the ratio between the number of alters in the same level of egos and the total number of alters using the following equation $similarity_s = \frac{n_s}{n}$ where $similarity_s$ is the quantified similarity between egos at level s in a given state, n_s is the number of alters at level s for a given state and n is the total number of alters. We calculated this ratio for each dynamic network of participants

and considered the levels of egos and alters at the same time. Then, we took the average of all calculated ratios. In all cases, we found that the percentage of similar alters does not exceed 50% which means that interaction between people does not necessarily take place based on homophily (Check Table D). We repeated the same process to quantify the similarity between people with respect to the number of interaction. We calculated the ratio between the number of interactions with alters in the same level of egos and the total number of interactions using the following equation $similarity_s = \frac{i_s}{i}$ where $similarity_s$ is the quantified similarity between egos at level s in a given state, i_s is the total interaction with alters at level s for a given state and i is the total number of interactions with alters. Again, we calculated this ratio for each dynamic network of participants and considered the levels of egos and alters at the same time. Then, we took the average of all calculated ratios. Again, we found that the percentage of similar alters does not exceed 50% (Check Table D).

2.2 Social Influence Processes

In our results, we identified four social influence processes : (1) attraction (2) repulsion (3) inertia (4) push.

2.2.1 Attraction and Repulsion

Formally, let $P(X \rightarrow Y|K)$ be the probability of transition $X \rightarrow Y$ conditional on intensity of contacts K with alters in level Z of a state S:

Attraction by K on egos in X level iff, for X different from Y, $P(X \rightarrow Y|K)$ is increasing with K and either $Z<X$ and $Y < X$, or $Z > X$ and $Y > X$.

Repulsion we have repulsion by K on people in X iff, for X different from Y, $P(X \rightarrow Y|K)$ is decreasing with K and either $Z < X$ and $Y < X$, or $Z > X$ and $Y > X$. Equivalently, we have repulsion by K on people in X iff, for X different from Y, $P(X \rightarrow Y|K)$ is increasing with K and either $Z < X$ and $Y \geq X$ or $Z > X$ and $Y \leq X$

Fig. D exemplifies some ways in which attraction and repulsion can manifest. We already gave examples of Case 1 in the main paper. Concerning Case 2, it was observed with agreeableness state and transition $(L \rightarrow N)$: the intensity of contacts with alters in the high level is associated with egos scoring high in the trait to upgrade the level from low to neutral.

Case 3 is exemplified by transition $(H \rightarrow L)$ of conscientiousness state. Intensity of contacts with alters in the neutral level is associated with shifting egos who have high trait score to the low level.

Case 4 is exemplified by transition $(N \rightarrow N)$ of conscientiousness. Intensity of contacts with alters in the low level is associated with increased probabilities of that stability of egos with high scores in the trait. All detailed examples are provided in the supporting information.

State	Number of Alters		Total Interaction	
**Extroversion**	**Mean**	**Standard Deviation**	**Mean**	**Standard Deviation**
Low	0.28	0.38	0.266	0.4
Neutral	0.49	0.41	0.199	0.366
High	0.28	0.38	0.49	0.45
**Conscientiousness**	**Mean**	**Standard Deviation**	**Mean**	**Standard Deviation**
Low	0.34	0.4	0.335	0.43
Neutral	0.44	0.39	0.42	0.45
High	0.3	0.388	0.29	0.417
**Agreeableness**	**Mean**	**Standard Deviation**	**Mean**	**Standard Deviation**
Low	0.29	0.35	0.31	0.41
Neutral	0.45	0.41	0.45	0.45
High	0.27	0.37	0.29	0.422
**Emotional Stability**	**Mean**	**Standard Deviation**	**Mean**	**Standard Deviation**
Low	0.35	0.39	0.36	0.44
Neutral	0.43	0.39	0.43	0.44
High	0.39	0.4	0.38	0.44
**Creativity**	**Mean**	**Standard Deviation**	**Mean**	**Standard Deviation**
Low	0.3	0.38	0.31	0.42
Neutral	0.54	0.4	0.55	0.45
High	0.346	0.4	0.344	0.445
**High PA (HPA)**	**Mean**	**Standard Deviation**	**Mean**	**Standard Deviation**
Low	0.38	0.4	0.387	0.44
Neutral	0.21	0.36	0.22	0.39
High	0.5	0.42	0.5	0.45
**Low NA (LNA)**	**Mean**	**Standard Deviation**	**Mean**	**Standard Deviation**
Low	0.27	0.37	0.25	0.4
Neutral	0.48	0.4	0.51	0.45
High	0.4	0.39	0.43	0.44

Table D: **Quantifying similarity between people according to their states.** We quantified the similarity between egos and alters in terms of their state levels. We calculated the ratio between the number of alters in the same level of egos and the total number of alters. Then, we took the mean and the standard deviation of those quantities. Also, we calculated the ratio between the total number of interactions with alters in the same level of egos and the total number of interactions. Then, we took the mean and the standard deviation of those quantities. In both cases, the similarity does not exceed 50%.

2.2.2 Inertia and Push

Formally, let P($X \to Y|K$) be the probability of transition $X \to Y$ conditional to contacts with people K in level X of a state S:

Inertia : K is inertial for people in level X iff $P(X \to X|K)$ increases with K. Equivalently, X is inertial iff, for X different from Y, $P(X \to Y|K)$ decreases with K.

Push : K pushes away egos in level X iff, for X different from Y, $P(X \to Y|X)$ increase with K. Equivalently, K pushes away alters in level X iff $P(X \to X|K)$ decreases with K.

Fig. E depicts the inertia and push influences triggered by intensity contacts with alters in egos' initial levels.

2.3 Control Variables

We believe that there might be diurnal rhythms in the between-subject correlations observed in our data [3]. Hence, transitions between levels of states can be attributed to the day of the week or the time of the day and not only attributed to social-situational factors and dispositional traits. To test for the existence of diurnal rhythm, we ran an ANOVA to check if the average level of each personality and affect measure changes systematically across days of the week or times of the day. Then, we controlled for the statistically significant diurnal rhythms by running regressions which controls for those rhythms.

Analysis of variance (ANOVA) is used to analyze the differences between group means. In our case, the groups contain either the days of the week or the times of the day (morning, midday and afternoon). In the case of week days, we found that there are no statistically significant differences of affect and personality states between weekdays except for creativity (p-value 0.00006). We took one more step to pairwise compare means of affect and personality states using Tukey's test [12]. Tukey HSD is a statistical test that is used with an ANOVA to do pairwise comparisons between the means of different treatments. In our case, we have seven weekdays and thus we have 10 pairwise comparisons. We found that people tend to be more creative at the end of the week than the beginning of the week: (1) Monday-Friday (diff: -0.23, p-value: 0.003) (2) Thursday-Friday (diff: 0.27, p-value: 0.0001). Also, there is a difference in the mean of creativity between Wednesday and Thursday (diff: -0.19, p-value: 0.018). If we were addressing transitions in creativity states between days, then controlling for the day of the week would be essential. However, we address transitions between times of the day. Therefore, the days of the week are not suitable to be used as control variables in our model.

When we ran the ANOVA test to investigate whether the means of personality and affect states changes systematically across times of the day, we found that those differences are statistically significant for most of the states. Therefore, we ran Tukey's test to check for pairwise comparisons across different times of the day. With respect to extraversion, people are less extrovert on average at the end of the day in comparison to their extraversion state's mean score at the beginning of the day (diff: -0.136, p-value: 0.029) or at midday (diff: -0.2, p-value: 0.0003). With respect to agreeableness, people got less agreeable on average in comparison to their mean score at the

beginning of the day (-0.113, p-value: 0.023). With respect to conscientiousness, people got less conscientious on average at midday in comparison to their mean score at the beginning of the day (diff: -0.2, p-value: 0.0000006) and they are less conscientious on average at the end of the day in comparison to their score at the beginning of the day (diff: -0.118, p-value: 0.016). However, people are more conscientious on average at the end of the day in comparison to their mean score at midday. With respect to emotional stability, people are less emotionally stable on average at the end of the day in comparison to midday (diff: -0.117, p-value: 0.033) and beginning of the day (diff: -0.14, p-value: 0.0078). Creativity on average doesn't change according to the time period. With respect to high positive affect state, people's mean score is lower at midday in comparison to the beginning of the day (-0.177, p-value = 0). Also, their mean score in HPA is lower at the end of the day in comparison to the beginning of the day. However, people's mean score at the end of the day is higher at the end of the day than the mean score at midday (diff: 0.088, p-value). With respect to low negative affect state, people's mean score is lower at the end of the day in comparison to their mean score at midday (diff: -0.099, p-value: 0.018) and the mean score at the beginning of the day (diff: -0.095, p-value: 0.0245). In our model, we address the transitions in states' level from mornings to middays and from middays to afternoons. Thus, we are interested in the mean differences in states in those two types of transitions.

We added the time of day as a diurnal control variable in our model besides the social-situational factors and dispositional traits to capture between-subject effect. We are interested to know the effect of the time period to which the transition takes place: midday (second surveys) in case of transitions from mornings to middays and the afternoon and afternoons (third surveys) in case of transitions from middays to afternoons. We represent the time of day as a dummy variable: 0 for middays (reference group) and 1 for afternoons.

First, we present the effects of time of the day and the interaction between time of the day and the corresponding trait. The coefficients of time of the day and the interactions are reported in tables E, F , G, H, I, J and K.

2.3.1 Effect of Time of Day

Extraversion The spontaneous transition (N $\rightarrow$ L and H $\rightarrow$ L) during the afternoon (transition from midday to afternoon) increases with respect to midday (transition from morning to midday). This is consistent with the post hoc comparisons of ANOVA that are reported earlier in this section. That is, the spontaneous tendency is for people who behaved more extravertedly at middays to behave introvertedly in afternoons.

Conscientiousness During the afternoon, people tend to move from the low level of conscientiousness state to reach the high level (L $\rightarrow$ H). Also during the afternoon, the spontaneous transitions (N $\rightarrow$ H) increases with respect to midday. Quite generally, people who behaved low or neutral in conscientiousness in midday tend to behave in a highly conscientious way in the afternoon.

Emotional Stability During the afternoon, the spontaneous transition (N $\rightarrow$ L) increases with respect to midday. However, the spontaneous transition (H $\rightarrow$ L) decreases with respect to midday. Therefore, during the afternoon, emotional stability level tend to decrease by only one level (N $\rightarrow$ L).

Creativity During the afternoon, people tend to move from the neutral level to the high level (N $\rightarrow$ H) with respect to midday. On the other hand, during the afternoon, the spontaneous transition (H $\rightarrow$ N) decreases with respect to midday.

High Positive Affect During the afternoon, people move from the low level to the high level (L $\rightarrow$ H) with respect to midday. This is consistent with ANOVA results that were reported earlier for HPA.

Low Negative Affect During the afternoon, spontaneous transitions (N $\rightarrow$ L) and (H $\rightarrow$ N) increases with respect to midday.

2.3.2 Interaction between Time of Day and Trait

We have anticipated that there would be an interaction between the corresponding trait and the time of the day. Therefore, we added the interaction as an additional control variable in our model.

With respect to extraversion, the baseline probablity of $(N \rightarrow N)$ stability for extroverts (according to trait level) increases from midday to the afternoon. However, the baseline probability of the same stability for introverts decreases during the same period. The probability of spontaneous $(H \rightarrow L)$ tranisition increases for all egos from midday to the afternoon and more markedly so for extroverts.

With respect to agreeableness, the baseline probablity of $(N \rightarrow N)$ stability for people with high score in agreeableness trait decreases from midday to the afternoon. However, the baseline probability of the same stability for people with low scores in agreeableness trait increases during the same period.

With respect to emotional stability, the baseline probablity of $(L \rightarrow H)$ transition for people with high score in emotional stability trait decreases from midday to the afternoon. However, the baseline probability of the same transition for people with low scores in emotional stability trait increases during the same period. The basline probability of $(N \rightarrow L)$ transitions importantly increase from midday to afteroon for neurotic people (according to trait) and decrease for emotionally stable ones. Putting this together with the former, it seems that emotionally stable people are indeed more stable because they have a strong tendency to maintain their level they had in the morning. The spontaneous transition $(H \rightarrow L)$ for emotionally stable people (according to the trait score) decreases. This confirms that emotionally stable people (according to trait) have decreasing probability of moving from where they are from midday to the afternoon while neurotic people tend to change their level more often.

With respect to creativity, the spontaneous $(L \rightarrow H)$ transition decreases in the afternoon for highly creative people (according to their trait). The opposite holds for people with low scores in

the creativity trait.

With respect to high positive affect state, the probability of spontaneous ($N \to N$) stability increases for people who have low scores in the trait in the afternoon and decreases for people who have high scores in the trait.

With respect to low negative affect state, the probability of spontaneous ($N \to N$) stability ncreases for people who have high scores in the trait in the afternoon and decreases for people who have low scores in the trait.

Second, we present our results of social influences in an increasing order according to provided details. First, we present the social influences of interaction between the social-situational factors and the dispositional factor. Second, we present the results from which we derive the social influences by reporting the direction of impact of each interaction between the two aforementioned factors: either increasing or decreasing the probability of the transition between each two levels of the state. Third, we present the coefficients of main effects and the interaction effects as a result of running our logistic regression model.

2.4 State-Specific Patterns

In Fig. F, we demonstrate the role that social-situational factors (intensity of contacts with alters in a certain level) played with respect to the dispositional factor (trait's level of the ego) towards the transitions between levels of the state in question.

Extraversion:

- Generally, alters acting introvertedly (according to state level) attract egos to stay or move towards their level except the egos who are already in the neutral state level and have high scores in the trait.
- Alters in the neutral level urge egos to act according to their traits.
- Surprisingly, alters in the high level of the state mostly attract egos with low trait scores to stay in the high level or move towards the alters' level whereas those alters repulse egos with high trait scores.

Agreeableness:

- Generally, alters in the low level of the state encourage egos to be less agreeable except for egos who are agreeable by nature (trait) and already in the high level.
- Alters in the neutral level urge egos with high trait scores to stay at their transient states while they mostly have an inconsistent role in case of egos with low trait scores.
- Alters in the high level play a balancing role. They encourage egos with low trait scores to stay at their level but they repulse those egos if they are in the low level. Inversely, they push egos with high trait scores who are in their level but they attract those egos if they are in the low level of the state.

Conscientiousness:

- Alters in the low level of the state attract egos to stay in the low level or switch to lower levels if those egos are already not conscientious by nature. Nevertheless, the alters cannot drag conscientious egos by nature towards their level unless the egos are already in the low level.
- Alters in the neutral level repulse egos to stay in their transient levels.
- Alters in the high level help egos to stay in the high level or move to their level except egos who are in the neutral level and have low trait scores.

Emotional Stability:

- Alters in the low level repulse egos who are in the neutral level.
- Alters in the neutral level of the state repulse egos, if those egos are in the low level of the state and have high scores in the trait.
- Mostly, alters in the high level repulse egos with low trait scores and enocurage egos to stay at the high level if the egos already have high trait scores.

Creativity:

- Alters in the low level mostly encourage egos to stay at their level.
- Alters in the neutral level mostly repulse egos away from their level except the egos who are in their level and have high trait scores.
- Alters in the high level mostly encourage egos to stay or move towards their level regardless of egos' traits.

High Positive affect (HPA):

- Surprisingly, alters in the low level push egos with low trait scores away from their level and attract egos with high scores in the trait to stay or move towards the low level.
- Alters in the neutral level push egos with low trait scores away from their level and encourage egos with high trait scores to stay at the neutral level
- Alters in the high level push egos with low trait scores away from the high level and attract egos with high trait scores to stay at the high level of the state.

Low Negative affect (LNA):

- Mostly, alters in the low level attract egos to stay or move to their level except the egos who are in the high state level and have low trait scores
- Alters in the neutral level mostly repulse egos away from their level.
- Surprisingly, alters in the high level attract egos with low trait scores and repulse egos with high trait scores.

2.5 State Diagrams of Dynamic States

In order to derive the social influences depicted in Fig. F, we observed the individual effects of the interaction between social-situational factors (intensity of contacts with alters in each level) and the dispositional factor (trait level of the ego) for each transition in the state in question as illustrated in Fig. G, Fig. H, Fig. I, Fig. J, Fig. K, Fig. L and Fig. M. In the figures, we labeled each arrow between each pair of levels of the state according to the direction of the effect of the mentioned factors (increasing (↑) or decreasing the probability of transition (↓)) We reported only the effects of factors that have statistically significant results in a given transition. Those effects could be: (1) the marginal effects of intensity of contacts with alters in a certain level of the state (e.g. intensity of contacts with alters in the high level) (2) the interaction between social-situational and dispositional factors e.g. the interaction between intensity of contacts with alters in the high level and the trait's high level of the ego.

2.5.1 Extraversion

$L \rightarrow L$ The intensity of contacts with alters in the neutral level is associated with an increase in the probability that the ego will stay at the low level ($L \rightarrow L$), in case the ego has a low score in the extraversion trait. The intensity of contacts with the same alters turns to be associated with a decrease in the probability of ($L \rightarrow L$), if the ego has a high score in the corresponding trait. This manifests the interaction effect of the ego's trait level and the state's levels of alters with whom the ego get in touch with.

$L \rightarrow H$ The intensity of contacts with alters in low and high levels of the state is associated with a decrease in the probability of this transition.

$N \rightarrow L$ The intensity of contacts with alters in the high level is associated with a decrease in the probability of ($N \rightarrow L$), if the ego has a low score in the trait. The intensity of contacts with the same alters is associated with an increase in the probability of the same transition, if the ego has a high score in the trait.

$N \to N$ The intensity of contacts with alters in the low level is associated with an increase in the probability of stability in the neutral level ($N \to N$) if the ego has a high score in the trait. However, the same intensity is associated with a decrease in the probability of ($N \to N$), if the ego has a low score in the trait.

$H \to L$ The intensity of contacts with alters in the high level is associated with a decrease in the probability of the transition, if the ego has a low score in the trait. The intensity of contacts with alters in the high level is associated with an increase in the probability of the transition, if the ego has a high score in the trait.

$H \to H$ The intensity of contacts with alters in the neutral level is associated with a decrease in the probability of the stability, if the ego has a low score in the trait. However, the intensity of contacts with the same alters becomes associated with an increase in the probability of the stability, if the ego has a high score in the trait. The intensity of contacts with alters in the high level is associated with an increase in the probability of the stability, if the ego has a low score in the trait. However, the intensity of contacts with alters in the high level of the state is associated with a decrease in the probability of the stability, if the ego has a high score in the trait.

2.5.2 Agreeableness

$L \to L$ The intensity of contacts with alters in the high level is associated with an increase in the probability of the stability, if the ego has a low score in the trait. However, the intensity of contacts with alters in the high level is associated with a decrease in the probability of the stability, if the ego has a high score in the trait.

$L \to N$ The intensity of contacts with alters in the high level of the state is associated with an increase in the probability of the transition, if the ego has a high score in the trait. However, the intensity of contacts with the same alters is associated with a decrease in the probability of the transition, if the ego has a low score in the trait.

$L \to H$ The intensity of contacts with alters in low or high levels of the state is associated with a decrease in the probability of the transition. Also, the intensity of contacts with alters in the neutral level of the state is associated with a decrease in the probability of the transition, if the ego has a high score in the trait. However, the intensity of contacts with alters in the neutral level is associated with an increase in the probability of transition of egos with low trait scores

$N \to N$ The intensity of contacts with alters in the neutral level of the state is associated with a decrease in the probability of the stability, if the ego has a low score in the trait. If the ego has a high score in the trait, then the intensity of contacts with the same alters becomes associated with an increase in the probability of stability.

$N \rightarrow H$ The intensity of contacts with alters in the neutral level of the state is associated with an increase in the probability of the transition, if the ego has a low score in the trait. However, the intensity of contacts with the same alters is associated with a decrease in the transition, if the ego has a high score in the trait.

$H \rightarrow N$ The intensity of contacts with alters in low and neutral levels of the state is associated with an increase in the probability of the transition, if the ego has a low score in the trait. However, the intensity of contacts with the same alters is associated with a decrease in the probability of the transition, if the ego has a high score in the trait. The intensity of contacts with alters in the high level of the state is associated with an decrease in the probability of the transition, if the ego has a low score in the trait. However, the intensity of contacts with the same alters is associated with an increase in the transition of egos with high trait scores.

$H \rightarrow H$ The intensity of contacts with alters in low and neutral levels of the state is associated with a decrease in the probability of the stability of egos with low scores in the trait, whereas the intensity of contacts with the alters is associated with an increase in the probability of the stability of egos with high scores in the trait. The intensity of contacts with alters in the high level of the state is associated with an increase in the probability of the stability of egos with low scores in the trait, whereas the intensity is associated with a decrease in the probability of the stability of egos with high scores in the trait.

2.5.3 Conscientiousness

$L \rightarrow L$ The intensity of contacts with alters in the neutral level is associated with an increase in the probability of staying at the low level. Also, the intensity of contacts with alters in the low level is associated with an increase in the probability of stability of egos with high trait scores. The intensity of contacts with alters in the high level is associated with a decrease in the probability of stability of egos with high trait scores.

$L \rightarrow N$ The intensity of contacts with alters in the neutral level is associated with a decrease in the probability of the transition.

$L \rightarrow H$ The intensity of contacts with alters in the neutral level is associated with a decrease in the probability of the transition of egos with high trait scores. Also, the intensity of contacts with alters in the low level is associated with a decrease in the probability of the transition of all egos.

$N \rightarrow L$ The intensity of contacts with alters in the low level is associated with a decrease in the probability of the transition of egos with high trait scores. However, the intensity of contacts with the same alters becomes associated with an increase in the probability of the transition of egos with low trait scores. The intensity of contacts with alters in the high level of the state is associated with a decrease in the probability of the transition of egos with high trait scores. However, the

intensity of contacts with the same alters becomes associated with an increase in the probability of the transition of egos with low trait scores.

$N \to N$ The intensity of contacts with alters in low and high levels of the state is associated with a decrease in the probability of the stability of egos with low traits scores.The intensity of contacts with the same alters is associated with an increase in the probability of the stability of egos with high trait scores.

$H \to L$ The intensity of contacts with alters in the high level is associated with a decrease in the probability of the transition. The intensity of contacts with alters in the low level is associated with an increase in the probability of the transition of egos with low trait scores.

$H \to N$ The intensity of contacts with alters in the neutral level of the state is associated with a decrease in the probability of the transition.

$H \to H$ The intensity of contacts with alters in the neutral level of the state is associated with an increase in the probability of the stability of egos with low trait scores.

2.5.4 Emotional Stability

$L \to H$ The intensity of contacts with alters in the neutral level of the state is associated with a decrease in the probability of the transition of egos with high trait scores.

$N \to L$ The intensity of contacts with alters in the low level of the state is associated with a decrease in the probability of the transition.

$N \to N$ The intensity of contacts with alters in the low level of the state is associated with an increase in the probability of the stability. Also, the intensity of contacts with alters in the high level of the state is associated with an increase in the probability of the stability of egos with low trait scores.

$N \to H$ The intensity of contacts with alters in the high level of the state is associated with a decrease in the probability of the transition of egos with low trait scores.

$H \to L$ The intensity of contacts with alters in the high level of the state is associated with a decrease in the probability of the transition of egos with high trait scores whereas the intensity of contacts is associated with an increase in the probability of the transition of egos with low trait scores.

2.5.5 Creativity

$L \to L$ The intensity of contacts with alters in the high level of the state is associated with a decrease in the probability of the stability.

$L \to H$ The intensity of contacts with alters in the low and neutral levels of the state is associated with a decrease in the probability of the transition. Also, the intensity of contacts with alters in the high level of the state is associated with a decrease in the probability of the transition of egos with high trait scores. However, the intensity of contacts with alters in the high level is associated with an increase in the probability of the transition of egos with low trait scores.

$N \to H$ The intensity of contacts with alters in the neutral level is associated with an increase in the probability of the transition of egos with low trait scores whereas the intensity becomes associated with a decrease in the probability of the transition of egos with high trait scores.

$H \to N$ The intensity of contacts with alters in the high level of the state is associated with a decrease in the probability of the transition.

2.5.6 High Positive Affect (HPA)

$L \to L$ The intensity of contacts with alters in the low level is associated with a decrease in the probability of the stability of egos with low trait scores. The intensity of contacts with the same alters becomes associated with an increase in the probability of the stability of egos with high trait scores.

$L \to H$ The intensity of contacts with alters in the low level is associated with a decrease in the probability of the transition of egos with high trait scores. The intensity of contacts with the same alters becomes associated with a increase in the probability of the transition of egos with low trait scores.

$N \to L$ The intensity of contacts with alters in the high levels is associated with an increase in the probability of the transition. The intensity of contacts with alters in the neutral level is associated with a decrease in the probability of the transition of egos with high trait scores. The intensity of contacts with the same alters becomes associated with a increase in the probability of the transition of egos with low trait scores.

$N \to N$ The intensity of contacts with alters in the neutral level is associated with a decrease in the probability of the stability of egos with low trait scores. The intensity of contacts with the same alters becomes associated with a increase in the probability of the stability of egos with high trait scores.

$H \to L$ The intensity of contacts with alters in the low level is associated with a decrease in the probability of the transition of egos with low trait scores whereas the intensity becomes associated with an increase in the probability of the transition of egos with high trait scores. The intensity of contacts with alters in the high level is associated with a decrease in the probability of the transition of egos with high trait scores whereas the intensity becomes associated with an increase in the probability of the transition of egos with low trait scores.

$H \to N$ The intensity of contacts with alters in the high level is associated with a decrease in the probability of the transition. The intensity of contacts with alters in the low level is associated with a decrease in the probability of the transition of egos with low trait scores whereas the intensity becomes associated with an increase in the probability of the transition of egos with high trait scores.

$H \to H$ The intensity of contacts with alters in the high level is associated with an increase in the probability of the stability of egos with high trait scores,whereas the intensity becomes associated with an decrease in the probability of the stability of egos with low trait scores. The intensity of contacts with alters in the low level is associated with an increase in the probability of the stability of egos with low trait scores,whereas the intensity becomes associated with an decrease in the probability of the stability of egos with high trait scores.

2.5.7 Low Negative Affect (LNA)

$L \to L$ The intensity of contacts with alters in the high level is associated with an increase in the probability of the stability of egos with high trait scores whereas the intensity becomes associated with a decrease in the probability of the stability of egos with low trait scores.

$L \to H$ The intensity of contacts with alters in neutral and low levels is associated with a decrease in the probability of the transition.

$N \to H$ The intensity of contacts with alters in the low level is associated with a decrease in the probability of the transition.

$H \to L$ The intensity of contacts with alters in the low level is associated with an increase in the probability of the transition of egos with high trait scores, whereas the intensity becomes associated with a decrease in the probability of the transition of egos with low trait scores.

$H \to N$ The intensity of contacts with alters in the neutral level is associated with an increase in the probability of the transition of egos with high trait scores, whereas the intensity becomes associated with a decrease in the probability of the transition of egos with low trait scores.

$H \rightarrow H$ The intensity of contacts with alters in the low level of the state is associated with an increase in the probability of the stability of egos with low trait scores whereas the intensity becomes associated with a decrease in the probability of the stability of egos with high trait scores.

3 Detailed Results

We report our detailed results (best sub-model according to QICC) for each transition in each state in Tables E, F , G, H, I, J and K. Each sub-table is labeled at the top with the corresponding transition in a given state. The mere effects of social-situational factors (intensity with alters in each level: L, N and H) and corresponding traits of egos (T) are reported in the table, if they are statistically significant. The interaction results between the two effect are reported also ($L * T$, $N * T$ and $H * T$), if they are statistically significant. The coefficients of the control variables are reported also: the main effect of the time of the day (period) and the interaction between the time of the day and the trait (period*T). Some reported coefficients are relatively small, therefore we used a threshold of 0.001 to consider them relevant. We focus more on the direction of the effect (increase or decrease in the probability) rather than the actual value of the effect.

**L to L**			**L to N**			**L to H**		
Variable	Coefficient	P-value	Variable	Coefficient	P-value	Variable	Coefficient	P-value
Intercept	-0.363	0.0016	Intercept	-0.218	0.0382	Intercept	-0.758	0.0000
N*T	-0.002	0.0011				H	-0.005	0.0000
						L	-0.005	0.0016

**N to L**			**N to N**			**N to H**		
Variable	Coefficient	P-value	Variable	Coefficient	P-value	Variable	Coefficient	P-value
Intercept	-1.508	0.0000	Intercept	0.177	0.0193	Intercept	-0.720	0.0000
N	0.0009	0.0155	T	-0.284	0.0382			
H*T	0.003	0.0100	H*T	-0.0009	0.0015			
(Period=1)	0.352	0.0258	L*T	0.002	0.0034			
			period*T	0.239	0.0065			

**H to L**			**H to N**			**H to H**		
Variable	Coefficient	P-value	Variable	Coefficient	P-value	Variable	Coefficient	P-value
Intercept	-1.503	0.0000	Intercept	-0.415	0.0002	H* T	-0.007	0.0001
(period=1)	0.792	0.0000				N*T	0.004	0.0006
T	-0.466	0.0042						
H*T	0.005	0.0008						
period*T	0.487	0.0112						

Table E: Extraversion Results

L to L			L to N			L to H		
Variable	Coefficient	P-value	Variable	Coefficient	P-value	Variable	Coefficient	P-value
Intercept	-0.350	0.003	H*T	0.007	0.004	Intercept	-1.033	0.0000
H*T	-0.007	0.001				L	-0.009	0.0000
						N	-0.007	0.0014
						H	-0.002	0.0020
						N*T	-0.011	0.0000

N to L			N to N			N to H		
Variable	Coefficient	P-value	Variable	Coefficient	P-value	Variable	Coefficient	P-value
Intercept	0.854	0.0000	Intercept	0.365	0.0000	Intercept	-1.472	0.000
			N*T	0.002	0.0187	N*T	-0.003	0.015
			(period=1) * T	0.205	0.0149			
			(period=0) * T	-0.170	0.0841			

H to L			H to N			H to H		
Variable	Coefficient	P-value	Variable	Coefficient	P-value	Variable	Coefficient	P-value
Intercept	-1.177	0.0000	Intercept	-0.282	0.053	N	-.004	.004
			H	-0.004	0.055	L*T	.005	.000
			L*T	-0.003	0.000	N*T	.008	.000
			N*T	-0.005	0.002	H* T	-.004	.001
			H*T	0.009	0.001			

Table F: Agreeableness Results

4 Broad Speculations and Further Work

So far, we have mainly been describing the data focusing on statistically significant effects and on their interpretation in terms of broader patterns of adaptation and complementarity. As stated in the main paper, we cannot make conclusive statements about causality without adopting additional assumptions. Nevertheless, in this section, we allow ourselves some liberty to make bolder speculations about possible implications of our findings, in case they corresponded to causal mechanisms. By definition, these observations are preliminary, and require further validation. Nonetheless, we believe they provide useful insights for further targeted studies.

4.1 Conscientiousness

Conscientious behaviors mutually reinforce: People acting conscientiously help each other maintain conscientious behavior, regardless of their trait.

Unconscientious behaviors mutually reinforce: As above but in the opposite direction, people acting unconscientiously help each other maintain the unconscientious behavior regardless of their trait.

L to L			L to N			L to H		
Variable	Coefficient	P-value	Variable	Coefficient	P-value	Variable	Coefficient	P-value
L	0.003	0.0017	Intercept	-0.441	0.0000	Intercept	-1.727	0.0000
H	-0.002	0.0129	N	-0.003	0.0000	(period=1)	0.715	0.0130
N	0.003	0.0000				L	-0.034	0.0000
T	-0.269	0.0286				N	-0.009	0.0041
L*T	0.003	0.0127				T	0.751	0.1131
H*T	-0.002	0.0060				L*T	0.013	0.0000
						N*T	-0.008	0.0012

N to L			N to N			N to H		
Variable	Coefficient	P-value	Variable	Coefficient	P-value	Variable	Coefficient	P-value
Intercept	-0.408	0.0005	H	-0.001	0.0447	Intercept	-1.564	0.00
L	-0.0025	0.0231	T	-0.406	0.0005	(period=1)	0.726	0.00
L*T	-0.006	0.0000	L*T	0.0025	0.0007	T	0.405	0.013
H*T	-0.002	0.0357	H*T	0.003	0.0010			

H to L			H to N			H to H		
Variable	Coefficient	P-value	Variable	Coefficient	P-value	Variable	Coefficient	P-value
Intercept	-1.579	0.0000	Intercept	-0.483	0.0002	N	0.003	0.0088
L	0.0024	0.0294	N	-0.0039	0.0004	T	0.5106	0.0000
H	-0.008	0.0255	T	-0.270	0.0050	N*T	-0.003	0.0144
L*T	-0.003	0.0006						

Table G: Conscientiousness Results

We cannot help you become good, but we can help you stay good: Conscientiously behaving people fail to pull bad apples. However if the latter already engage in virtuous behaviors, conscientiously behaving people can help them keep going.

We cannot make you bad, unless you already chose to: Similarly, unconscientiously behaving people fail to pull good apples. But if the latter are already going astray, the former can keep them on the wrong way.

4.2 Agreeableness

The spiral of stubbornness: If someone is behaving stubbornly (e.g. being difficult, argumentative, less agreeable), then interacting with people who are behaving similarly helps keep him/her in that state irrespective of his/her trait.

stubbornly behaving people activate others' stubbornness: If you are stubborn by nature, and happen to behave agreeably, stay away from argumentative people, because they can stimulate your natural stubbornness.

L to L			L to N			L to H		
Variable	Coefficient	P-value	Variable	Coefficient	P-value	Variable	Coefficient	P-value
Intercept	0.273	0.0252	Intercept	-0.428	0.0001	Intercept	-2.451	0.0000
						N	-0.005	0.0208
						T	1.102	0.0079
						T*N	-0.006	0.0097
						(period=1) * T	-3.001	0.0000

N to L			N to N			N to H		
Variable	Coefficient	P-value	Variable	Coefficient	P-value	Variable	Coefficient	P-value
Intercept	-1.005	0.000	L	0.001	0.008	Intercept	-1.001	0.000
(period=1)	0.548	0.002	H	0.005	0.000	H	-0.012	0.002
L	-0.002	0.000	T	0.379	0.001	H*T	0.011	0.002
T	0.271	0.056	H*T	-0.007	0.000			
(period=1) * T	-0.907	0.002						

H to L			H to N			H to H		
Variable	Coefficient	P-value	Variable	Coefficient	P-value	Variable	Coefficient	P-value
Intercept	-1.398	0.000	Intercept	-0.690	0.000	Intercept	0.325	0.044
(period=1)	-0.503	0.006						
H	-0.014	0.034						
H*T	-0.018	0.021						
(period=1) *T	-0.771	0.014						
(period=0) *T	0.035	0.890						

Table H: Emotional Stability Results

4.3 Extraversion

Bringing you out of the shell: If you are introvert by nature, hanging out with people behaving extrovertedly can help you become more outgoing.

Stealing the thunder effect: If you are extrovert by nature, and acting extrovertedly in a group of extroverts, the latter might ”steal your thunder” and suppress your extroverted behavior.

4.4 Creativity

Creative behaviors are attractive and mutually reinforce: If you want to behave or continue behaving in a creative way, hang out with creative people.

Low-creative behaviors mutually reinforce: People behaving uncreatively help each other maintain the uncreative behavior, regardless of their trait.

L to L			L to N			L to H		
Variable	Coefficient	P-value	Variable	Coefficient	P-value	Variable	Coefficient	P-value
H	-0.010	0.0009	Intercept	-0.115	0.1284	Intercept	-1.125	0.0001
						L	-0.0432	0.0013
						N	-0.009	0.0001
						T	1.910	0.0000
						L*T	-0.022	0.0019
						N*T	-0.0067	0.0001
						H*T	-0.027	0.0000
						(period=1) * T	-1.085	0.0025

N to L			N to N			N to H		
Variable	Coefficient	P-value	Variable	Coefficient	P-value	Variable	Coefficient	P-value
Intercept	-0.916	0.0000	Intercept	0.318	0.0000	Intercept	-1.53	0.0000
						(period=1)	0.466	0.009
						T	0.689	0.0000
						N*T	-0.002	0.003

H to L			H to N			H to H		
Variable	Coefficient	P-value	Variable	Coefficient	P-value	Variable	Coefficient	P-value
Intercept	-1.268	0.0000	H	-0.026	0.0000	T	0.533	0.0012
			T	-0.728	0.0000			
			H*T	0.013	0.0035			
			(period=1)	-0.660	0.0015			

Table I: Creativity Results

References

[1] Tamlin S Conner, Howard Tennen, William Fleeson, and Lisa Feldman Barrett. Experience sampling methods: A modern idiographic approach to personality research. *Social and personality psychology compass*, 3(3):292–313, 2009.

[2] Mihaly Csikszentmihalyi and Reed Larson. Validity and reliability of the experience-sampling method. *The Journal of nervous and mental disease*, 175(9):526–536, 1987.

[3] Scott A Golder and Michael W Macy. Diurnal and seasonal mood vary with work, sleep, and daylength across diverse cultures. *Science*, 333(6051):1878–1881, 2011.

[4] Samuel Gosling, Peter Rentfrow, and William Swann. A very brief measure of the big-five personality domains. *Journal of Research in Personality*, 37(6):504–528, 2003.

[5] A.L. Hill, D.G. Rand, M.A. Nowak, and N. Christakis. Emotions as infectious diseases in a large social network: the SISa model. *Proceedings of Royal Society B: Biological Sciences*, 277(1701):3827–3835, 2010.

L to L			L to N			L to H		
Variable	Coefficient	P-value	Variable	Coefficient	P-value	Variable	Coefficient	P-value
T	-0.424	0.0002	Intercept	-0.928	0.0000	Intercept	-1.027	0.0000
L*T	0.0013	0.0256				T	0.4947	0.0015
						L*T	-0.002	0.0000
						period	0.511	0.0005

N to L			N to N			N to H		
Variable	Coefficient	P-value	Variable	Coefficient	P-value	Variable	Coefficient	P-value
Intercept	-0.439	0.001	Intercept	-0.73	0	Intercept	-0.34	0.006
H	0.002	0.037	N	-0.005	0.0486			
N	0.007	0.00	N*T	0.0196	0.0704			
N*T	-0.015	0.05	(period=1) *T	-0.356	0.0198			
			(period=0)*T	0.015	0.9122			

H to L			H to N			H to H		
Variable	Coefficient	P-value	Variable	Coefficient	P-value	Variable	Coefficient	P-value
Intercept	-1.134	0.0000	Intercept	-1.180	0.0000	Intercept	0.416	0.0016
L	0.0011	0.0306	H	-0.0042	0.0016	L	-0.0013	0.0484
T	-0.440	0.0359	T	-0.448	0.0080	T	0.594	0.0002
L*T	0.002	0.0451	L*T	0.002	0.0249	L*T	-0.003	0.0019
H*T	-0.006	0.002				H*T	0.003387911	0.0014

Table J: High Positive Affect (HPA) Results

[6] Bruno Lepri, Jacopo Staiano, Giulio Rigato, Kyriaki Kalimeri, Ailbhe Finnerty, Fabio Pianesi, Nicu Sebe, and Alex Pentland. The sociometric badges corpus: A multilevel behavioral dataset for social behavior in complex organizations. In *SocialCom/PASSAT*, pages 623–628, 2012.

[7] Bruno Lepri, Jacopo Staiano, Giulio Rigato, Kyriaki Kalimeri, Ailbhe Finnerty, Fabio Pianesi, Nicu Sebe, and Alex Pentland. The sociometric badges corpus: A multilevel behavioral dataset for social behavior in complex organizations. In *Privacy, Security, Risk and Trust (PASSAT), International Confernece on Social Computing (SocialCom)*, pages 623–628. IEEE, 2012.

[8] Daniel Olguín Olguín, Benjamin N Waber, Taemie Kim, Akshay Mohan, Koji Ara, and Alex Pentland. Sensible organizations: Technology and methodology for automatically measuring organizational behavior. *IEEE Transactions on Systems, Man, and Cybernetics, Part B (Cybernetics)*, 39(1):43–55, 2009.

[9] M. Perugini and Blas L. The big five marker scales (bfms) and the italian ab5c taxonomy: Analyses from an emic-etic perspective, 2012.

[10] Auke Tellegen and Niels G. Waller. Exploring personality through test construction: Development of the multidimensional personality questionnaire. In Gregory J. Boyle, Gerald

L to L

Variable	Coefficient	P-value
H*T	0.003	0.0215

L to N

Variable	Coefficient	P-value
Intercept	-0.157	0.1354

L to H

Variable	Coefficient	P-value
Intercept	-1.151	0.0000
L	-0.003	0.0059
N	-0.017	0.0000

N to L

Variable	Coefficient	P-value
Intercept	-1.156	0.0000
N*T	0.001	0.0481
period	0.615	0.0000

N to N

Variable	Coefficient	P-value
Intercept	0.440	0.0000
N	0.0005	0.0602
(period=1) *T	0.248	0.0265
(period=0) *T	-0.267	0.0567

N to H

Variable	Coefficient	P-value
Intercept	-1.317	0.0000
L	-0.009	0.0034
N	-0.0007	0.0305

H to L

Variable	Coefficient	P-value
Intercept	-1.193	0.0000
L	-0.002	0.0490
T	-0.594	0.0072
L*T	0.004	0.0004

H to N

Variable	Coefficient	P-value
Intercept	-0.876	0.0000
T	-0.403	0.0018
N*T	0.002	0.0601
period	0.403	0.0011

H to H

Variable	Coefficient	P-value
(period=1)	0.011	0.9501
(period=0)	0.321	0.0587
T	0.708	0.0000
L*T	-0.003	0.0228

Table K: Low Negative Affect (LNA) Results

Matthews, and Donald H. Saklofske, editors, *The SAGE Handbook of Personality Theory and Assessment: Volume 2 — Personality Measurement and Testing*, pages 261–293. SAGE Publications Ltd, 2008.

[11] David Watson, Lee A Clark, and Auke Tellegen. Development and validation of brief measures of positive and negative affect: the PANAS scales. *Journal of personality and social psychology*, 54(6):1063, 1988.

[12] Brian S Yandell. *Practical data analysis for designed experiments*, volume 39. 1997.

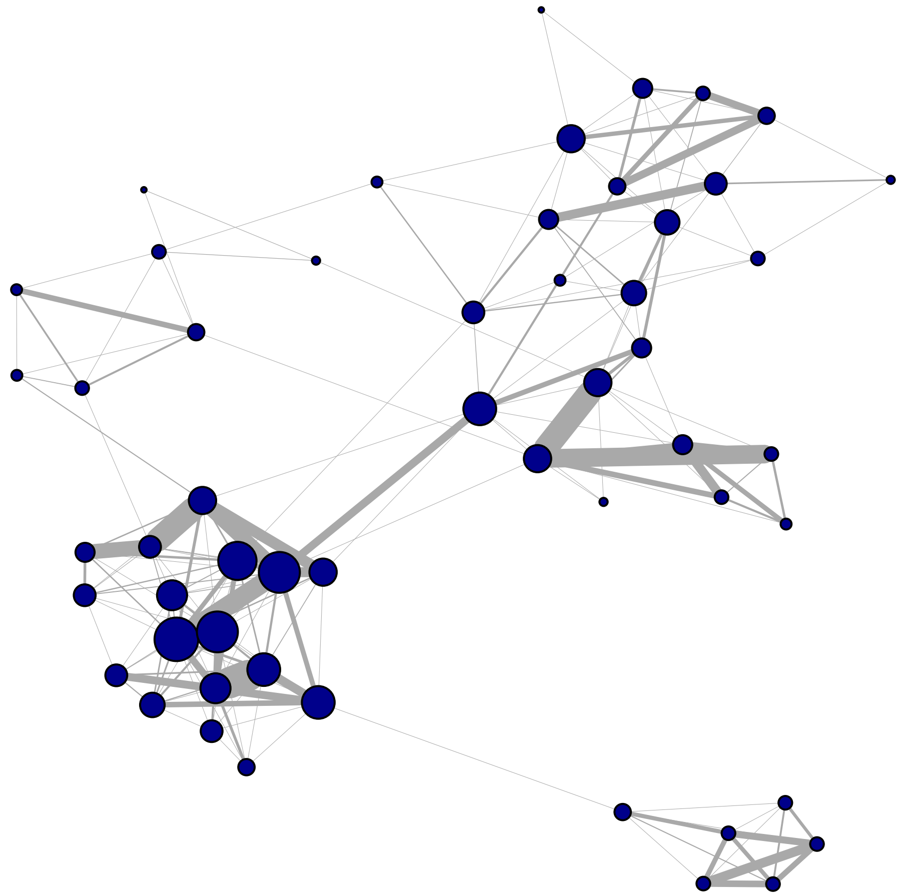

Figure A: **The composite social network of participants.** The aggregate social network is plotted showing the social ties among participants where the nodes are the participants and the edges are the IR hits detected between each pair of participants. The size of the nodes indicates their degree while the thickness of the edges is proportional number of total number of IR hits between a pair of participants. *Remarks: We considered only IR hits that are in total more than 10 to create the social ties between participants.*

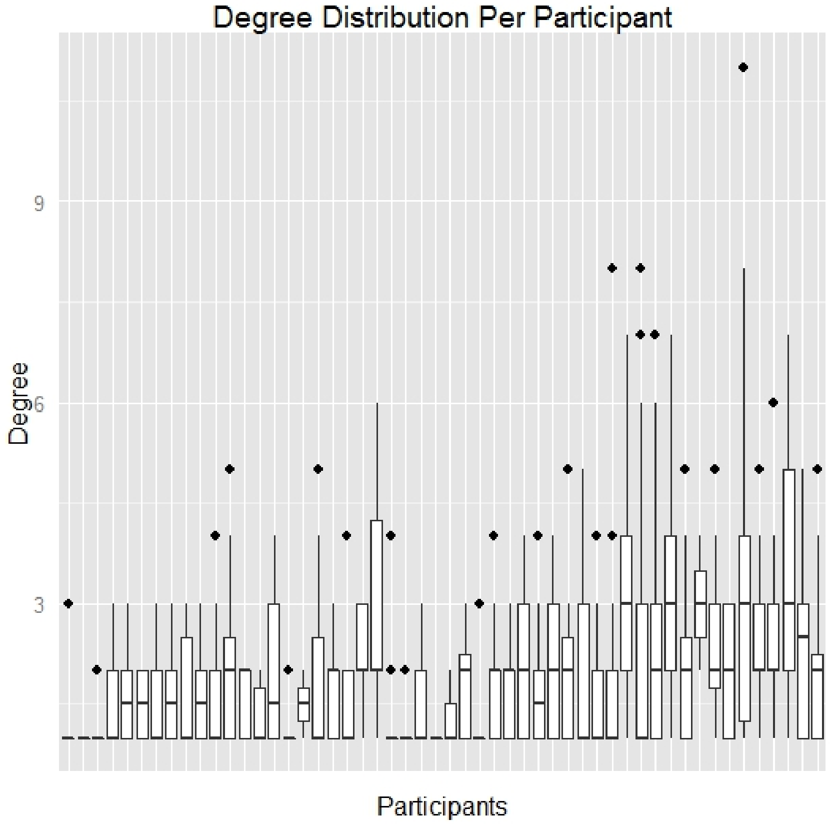

Figure B: **The Degree Distribution for each Participant** Each box plot shows the variation in the number of alters in dynamic networks of participants. We can observe that there is a variation in the number of alters in the dynamic networks of participants.

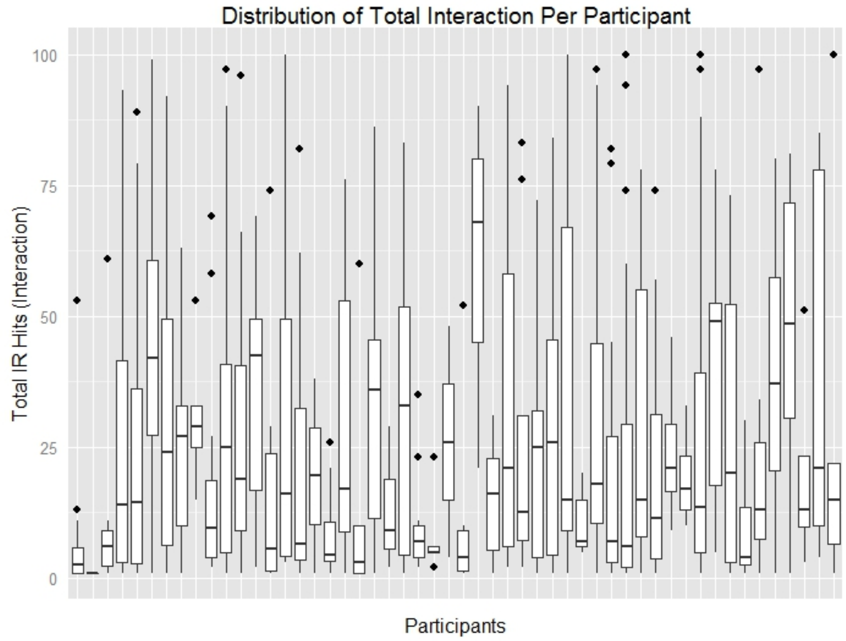

Figure C: **The Interaction Distribution for each Participant** Each box plot shows the variation in the total number of interactions (IR hits) in dynamic networks of participants. We can observe that there is a variation in the number of interactions in the dynamic networks of participants.

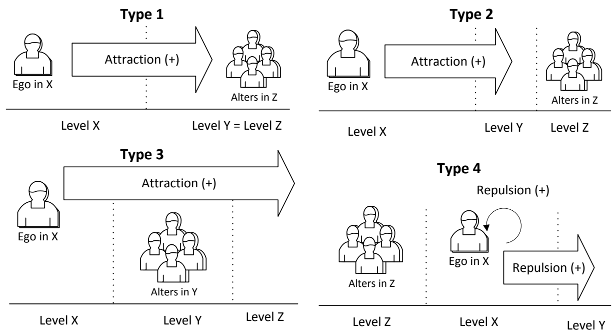

Figure D: Attraction and Repulsion. Case 1-3 exemplify different ways which attraction can manifest. In Case 1, the presence of alters in level Z is associated with ego's switching to that level. In Case 2, the ego moves to level Y which is though lower than that of the attracting alters but higher than his/her original level. In Case 3, the ego moves to a level that is higher of both his/her original one and that of the attracting alters. Case 4 exemplifies repulsion: the presence of alters in a lower level is associated with the ego remaining at his/her current state or moving to a higher level. Attraction to lower levels works in the same way.

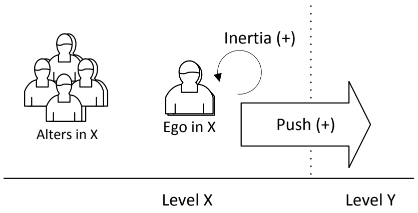

Figure E: Inertia and Push: Alters are inertial for egos to move away from the alters' levels by either lowering the transition probability to another level or increasing the probability of staying at the alter's level. Vice versa for the push affect

Extrav.	L		N		H	
Low	A(I)	A(I)	C(R)	A(T)	C(R)	C(R)
Neutral	A(T)	C(R)			A(T)	C(R)
High			A(T)	C(R)	A(I)	C(P)

Agr.	L		N		H	
Low	A(I)	A(I)	A(I)	C(R)	C(R)	A(T)
Neutral			C(P)	A(I)		
High	A(T)	C(R)	A(T)	C(R)	A(I)	C(P)

Consc.	L		N		H	
Low	A(I)	A(I)	C(R)	C(R)		A(T)
Neutral	A(T)	C(R)			C(R)	A(T)
High	A(T)		C(R)	C(R)	A(I)	A(I)

ES	L		N		H	
Low				C(R)		
Neutral	C(R)	C(R)			C(R)	
High					C(P)	A(I)

Creat.	L		N		H	
Low	A(I)	A(I)	C(R)	C(R)	A(T)	A(T)
Neutral			C(P)	A(I)		
High					A(I)	A(I)

HPA	L		N		H	
Low	C(P)	A(I)				
Neutral			C(P)	A(I)	C(R)	C(R)
High	C(R)	A(T)			C(P)	A(I)

LNA	L		N		H	
Low	A(I)	A(I)	C(R)	C(R)	A(T)	C(R)
Neutral	A(T)	A(T)				
High	C(R)	A(T)	C(R)	A(T)		

Legend

Intensity of contacts with alters in the low level of the state

Intensity of contacts with alters in the neutral level of the state

Intensity of contacts with alters in the high level of the state

Egos scoring low in the trait

Egos scoring high in the trait

Figure F: Social Influences: The table summarizes each state's results by means of the two adverse effects introduced in the text: adaptation (A) and complementarity (C). Also, the detailed effects are listed between the brackets: (1) attraction (T) (2) repulsion (R) (3) inertia (I) and (4) push (P). Rows represent ego's state levels; columns are labeled with alters' levels and sub-labeled with ego's trait level (Low or High). Cells report the effects observed when egos in the corresponding state level and trait level interact with alters in the corresponding state level.

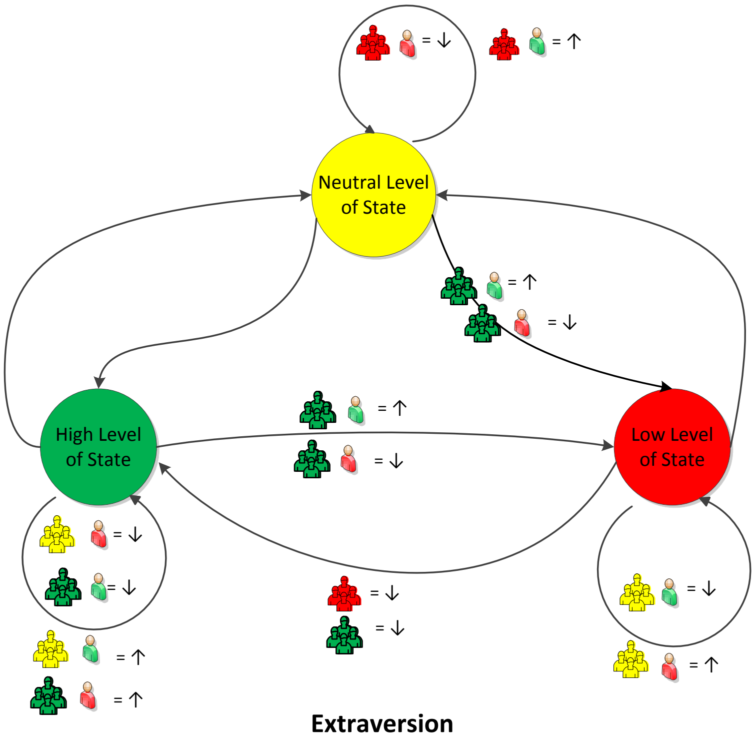

Figure G: Level transition graph for extraversion state: Nodes represent extraversion level of the ego. An arrow between two circles represents the transition from one level to another. These transitions are labeled with conditions that affect the corresponding probabilities. Icons represent the extraversion levels of alters and ego's trait level. Symbol ↑ (respectively ↓) indicates an increase (respectively decrease) in transition probability associated with the given combination of alters state level and ego trait level. For example, if the ego is in the low level of the extraversion state, then the probability of him/her transitioning to the high level decreases with his/her interactions with alters in the high level.

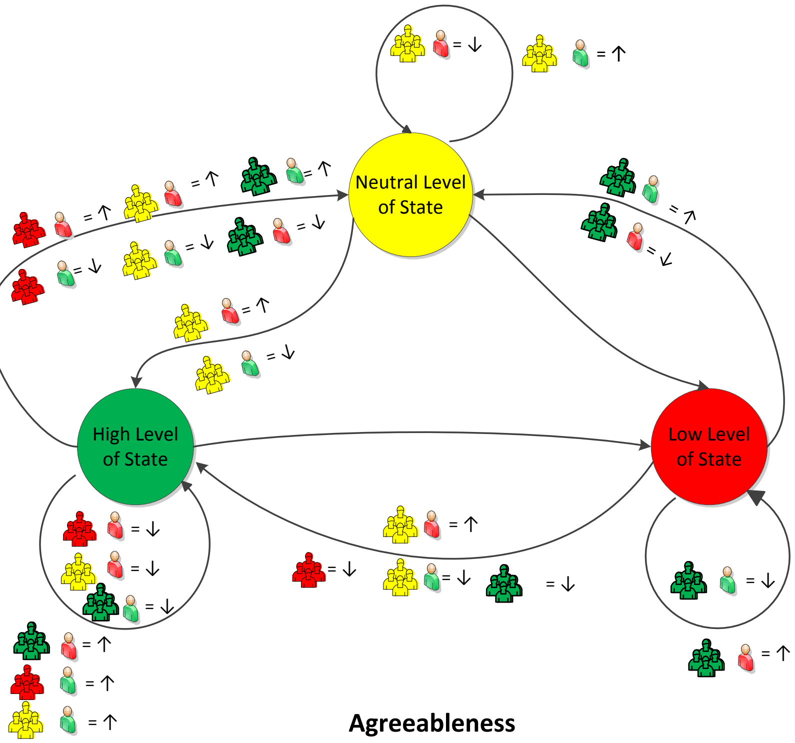

Figure H: Level transition graph for agreeableness state: Nodes represent agreeableness level of the ego. An arrow between two circles represents the transition from one level to another. These transitions are labeled with conditions that affect the corresponding probabilities. Icons represent the agreeableness levels of alters and ego's trait level. Symbol ↑ (respectively ↓) indicates an increase (respectively decrease) in transition probability associated with the given combination of alters state level and ego trait level. For example, if the ego is in the low level of the agreeableness state, then the probability of him/her transitioning to the high level decreases with his/her interactions with alters in the high level.

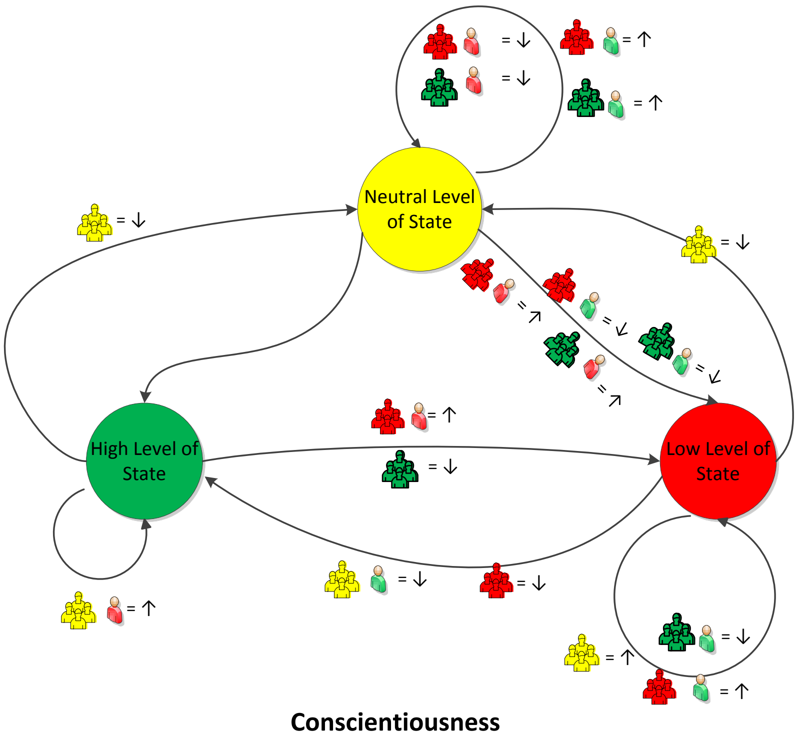

Figure I: Level transition graph for conscientiousness state: Nodes represent conscientiousness level of the ego. An arrow between two circles represents the transition from one level to another. These transitions are labeled with conditions that affect the corresponding probabilities. Icons represent the conscientiousness level of alters and ego's trait level. Symbol ↑ (respectively ↓) indicates an increase (respectively decrease) in transition probability associated with the given combination of alters state level and ego trait level. For example, if the ego is in the low level of the conscientiousness states, then the probability of him/her transitioning to the high level decreases with his/her interactions with alters in the high level.

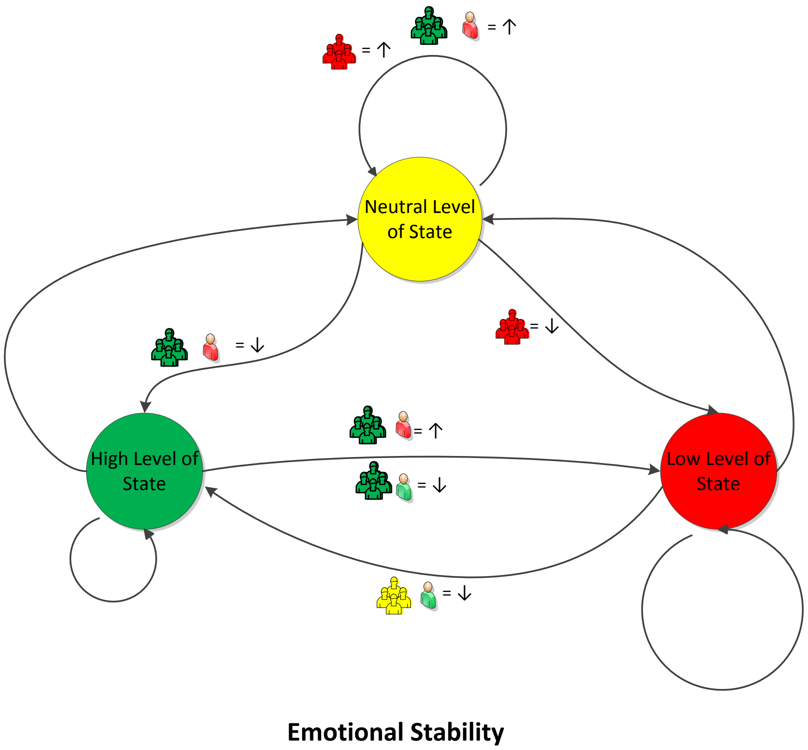

Figure J: Level transition graph for emotional stability state: Nodes represent emotional stability level of the ego. An arrow between two circles represents the transition from one level to another. These transitions are labeled with conditions that affect the corresponding probabilities. Icons represent the emotional stability level of alters and ego's trait level. Symbol ↑ (respectively ↓) indicates an increase (respectively decrease) in transition probability associated with the given combination of alters state level and ego trait level. For example, if the ego is in the low level of the emotional stability states, then the probability of him/her transitioning to the high level decreases with his/her interactions with alters in the high level.

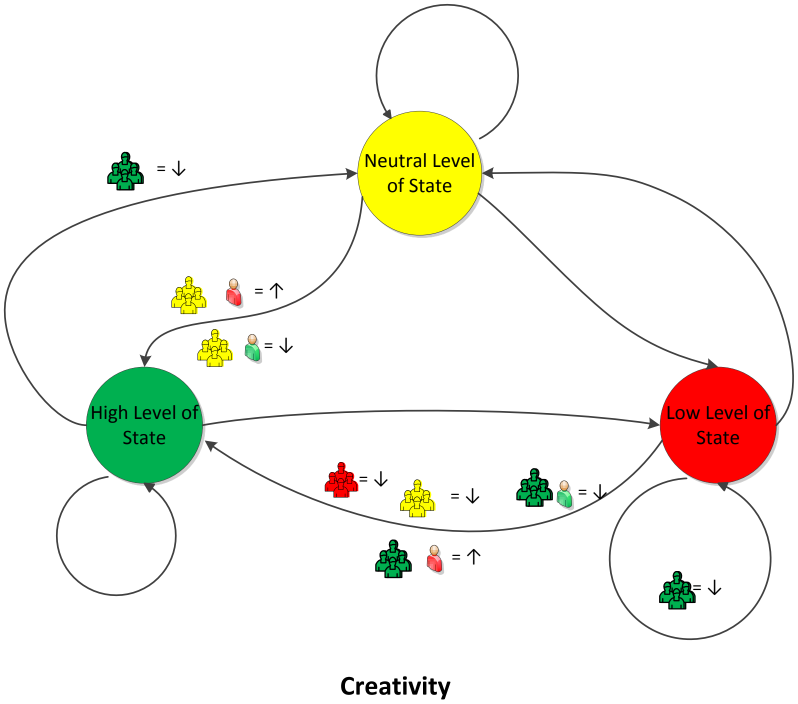

Figure K: Level transition graph for creativity state: Nodes represent creativity level of the ego. An arrow between two circles represents the transition from one level to another. These transitions are labeled with conditions that affect the corresponding probabilities. Icons represent the creativity levels of alters and ego's trait level. Symbol ↑ (respectively ↓) indicates an increase (respectively decrease) in transition probability associated with the given combination of alters state level and ego trait level. For example, if the ego is in the low level of the creativity state, then the probability of him/her transitioning to the high level decreases with his/her interactions with alters in the high level.

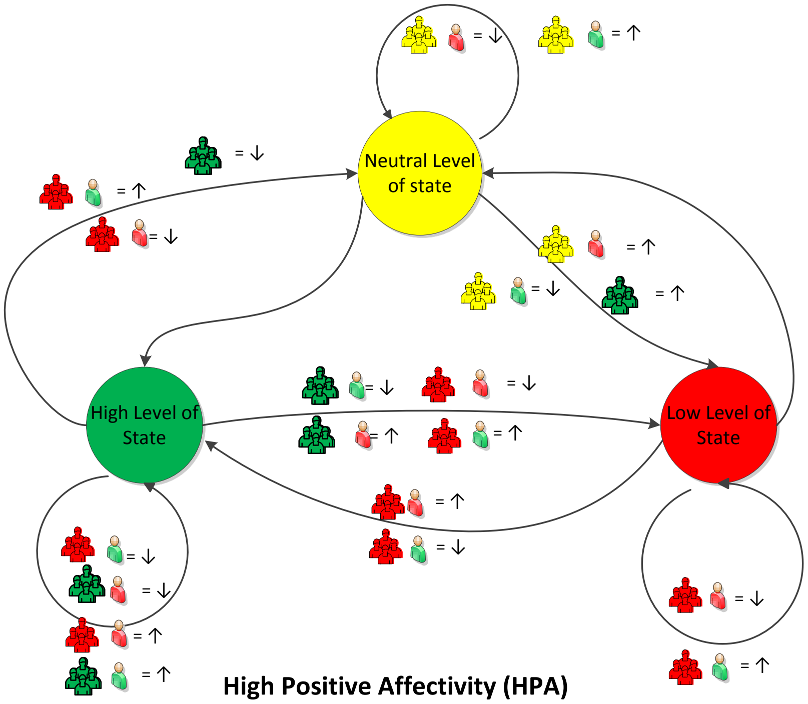

Figure L: Level transition graph for high positive affect state: Nodes represent high positive affect level of the ego. An arrow between two circles represents the transition from one level to another. These transitions are labeled with conditions that affect the corresponding probabilities. Icons represent the high positive affect levels of alters and ego's trait level. Symbol ↑ (respectively ↓) indicates an increase (respectively decrease) in transition probability associated with the given combination of alters state level and ego trait level. For example, if the ego is in the low level of the high positive affect state, then the probability of him/her transitioning to the high level decreases with his/her interactions with alters in the high level.

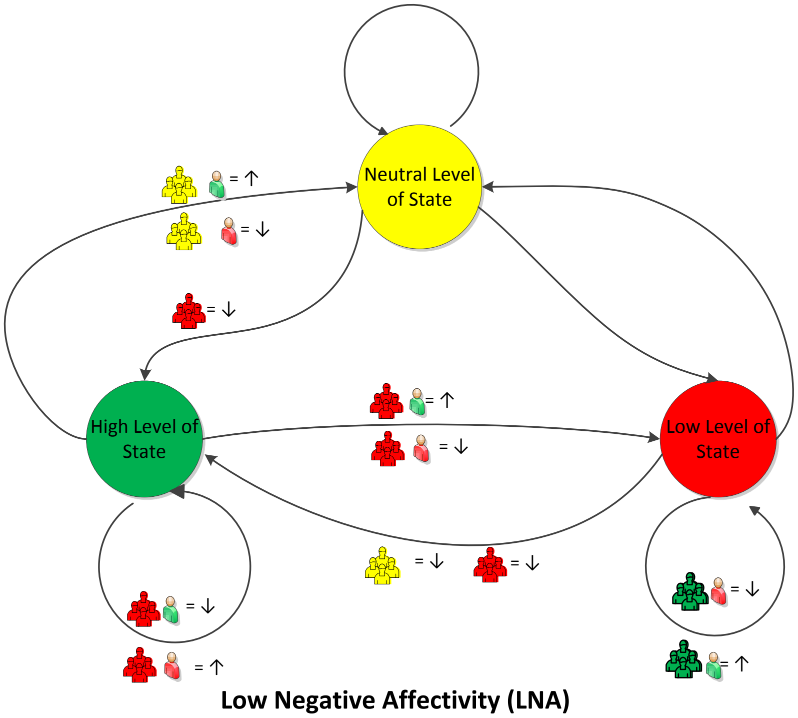

Figure M: Level transition graph for low negative affect state: Nodes represent low negative affect level of the ego. An arrow between two circles represents the transition from one level to another. These transitions are labeled with conditions that affect the corresponding probabilities. Icons represent the low negative affect level of alters and ego's trait level. Symbol ↑ (respectively ↓) indicates an increase (respectively decrease) in transition probability associated with the given combination of alters state level and ego trait level. For example, if the ego is in the low level of the low negative affect states, then the probability of him/her transitioning to the high level decreases with his/her interactions with alters in the high level.
